# Facilitating Heterogeneous Effect Estimation via Statistically Efficient Categorical Modifiers

**DOI:** 10.1080/01621459.2026.2635078

**Published:** 2026-05-21

**Authors:** Daniel R. Kowal

**Affiliations:** Department of Statistics and Data Science, Cornell University, Ithaca, NY

**Keywords:** Discrete data, Interactions, Penalized Estimation, Regression analysis

## Abstract

Categorical covariates such as race, sex, or group are ubiquitous in regression analysis. While main-only (or ANCOVA) linear models are predominant, linear models that include categorical-continuous or categorical-categorical interactions are increasingly important and allow heterogeneous, group-specific effects. However, with standard approaches, the addition of categorical interactions fundamentally alters the estimates and interpretations of the main effects, often inflates their standard errors, and introduces significant concerns about group (e.g., racial) biases. We advocate an alternative parameterization and estimation scheme using *abundance-based constraints* (ABCs). ABCs induce a model parameterization that is both interpretable and equitable. Crucially, we show that with ABCs, the addition of categorical interactions (a) leaves main effect estimates unchanged and (b) enhances their statistical power, under reasonable conditions. Thus, analysts can, and arguably *should* include categorical interactions in linear models to discover potential heterogeneous effects—without compromising estimation, inference, and interpretability for the main effects. Using simulated data, we verify these invariance properties for estimation and inference and showcase the capabilities of ABCs to increase statistical power. We apply these tools to study demographic heterogeneities among the effects of social and environmental factors on STEM educational outcomes for children in North Carolina. An R package lmabc is available. Supplementary materials for this article are available online, including a standardized description of the materials available for reproducing the work.

## 1. Introduction

Interactions are remarkably valuable in linear regression analysis. In particular, interactions between a categorical (or nominal) variable and a continuous or categorical variable—which we refer to as *cat-modifiers*, short for “categorical effect modifiers”—are crucial for discovering and quantifying heterogeneous effects. A prominent example is race: due to structural racism and discrimination, the effects of many important variables on health and life outcomes vary by race (Williams, Lawrence, and Davis 2019), with race often interacting with sex or socioeconomic status (Schoendorf et al. 1992; Bauer 2014). Cat-modifiers are also relevant for studying gene-environment interactions (Miao, Wu, and Lu 2024) and appear broadly in the social and behavioral sciences (Krefeld-Schwalb, Sugerman, and Johnson 2024).

Yet there are significant obstacles to the inclusion of cat-modifiers in linear regression analysis. Broadly, cat-modifiers alter the interpretation of the main effects, introduce concerns about equity across categorical groups (e.g., for race, sex, etc.), change the main effect estimates, and typically inflate the main effect standard errors (SEs). Consequently, cat-modifiers are often omitted or misreported (Knol et al. 2009), which falsely suppresses heterogeneity.

We argue that, *with the right parameterization*, cat-modifiers can and arguably *should* be included in linear regression models with categorical covariates. To establish ideas, suppose we have p continuous covariates $x={\left(x_{1},\ldots ,x_{p}\right)}^{⊤}$ and K categorical variables $C={\left(C_{1},\ldots ,C_{K}\right)}^{⊤}$ with $L_{k}$ levels for each categorical variable k=1,…,K. We consider regression models for data ${\left\{\left(x_{i},c_{i},y_{i}\right)\right\}}_{i=1}^{n}$ parameterized by a linear regression function μ(x,c) that typically models the conditional expectation E(Y∣x,c) or a transformed version for generalized linear models. We distinguish between two classes of linear models: those that do not include cat-modifiers and those that do. First, the *main-only* model includes multiple continuous and categorical variables, but no interactions:

(1) $$\mu^{M}(x,c)=\alpha_{0}^{M}+x^{⊤}\alpha^{M}+\sum_{k=1}^{K}\beta_{k,c_{k}}^{M}$$

where $c_{k}$ indexes the $L_{k}$ levels of the kth categorical variable. In Wilkinson notation, (1) is $\text{y}\sim {\text{x}}_{1}+\cdots +{\text{x}}_{p}+{\text{c}}_{1}+\cdots +{\text{c}}_{K}$. Second, the *cat-modified* model expands (1) to allow categorical-continuous and categorical-categorical interactions:

(2) $$\mu (x,c)=\alpha_{0}+x^{⊤}\alpha +\sum_{k=1}^{K}\beta_{k,c_{k}}+\sum_{k=1}^{K}x^{⊤}\gamma_{k,c_{k}}+\sum_{k=1}^{K-1}\sum_{k^{\prime }=k+1}^{K}\gamma_{k,k^{\prime },c_{k},{c_{k}}^{\prime }}$$

or equivalently, $\text{y}\sim \left({\text{x}}_{1}+\cdots +{\text{x}}_{p}\right)*\left({\text{c}}_{1}+\cdots +\right.\left.{\text{c}}_{K}\right)+{\text{c}}_{1}*{\text{c}}_{2}+\cdots +{\text{c}}_{K-1}*{\text{c}}_{K}$, using pairwise interactions for convenience. Our notation emphasizes that the parameters in (1) and (2) are fundamentally distinct, even though these models are nested.

The advantage of the cat-modified model is the ability to estimate heterogeneous, group-specific effects for each $x_{j}$. While both models specify *group-specific intercepts* (consider (1) and (2) with x=0), only the cat-modified model features *group-specific slopes*:

(3) $$\mu_{x_{j}}^{\prime }(c)≔\mu \left(x_{j}+1,x_{-j},c\right)-\mu \left(x_{j},x_{-j},c\right)=\alpha_{j}+\sum_{k=1}^{K}\gamma_{j,k,c_{k}}.$$

Heterogeneity arises because the effect of $x_{j}$ varies across different groups c. By comparison, this does not occur in the mainonly model: $\mu_{x_{j}}^{M^{\prime }}≔\mu^{M}\left(x_{j}+1,x_{-j},c\right)-\mu^{M}\left(x_{j},x_{-j},c\right)=\alpha_{j}^{M}$. The remaining difference between (1) and (2) is that the latter model also includes categorical-categorical interactions via $\gamma_{k,k^{\prime },c_{k},c_{k^{\prime }}}$, which impact the group-specific intercepts.

For concreteness, we consider two popular cases. Empirical examples are given in Tables 1 and E.1, respectively, and these cases are revisited subsequently.

### Example 1 (ANCOVA).

Suppose we have p=1 continuous variable x∈R and K=1 categorical variable race with $L_{R}$ groups. The main-only model (1) is then

(4) $$\mu^{M}(x,r)=\alpha_{0}^{M}+x\alpha_{1}^{M}+\beta_{r}^{M}$$

or equivalently, y~x+race, with group-specific intercepts, $\mu^{M}(0,r)=\alpha_{0}^{M}+\beta_{r}^{M}$ for each race group r, but a global (race-invariant) slope, $\mu_{x}^{M^{\prime }}=\alpha_{1}^{M}$. Thus, (4) produces parallel lines with race-specific vertical shifts. By comparison, the cat-modified model (2) is

(5) $$\mu (x,r)=\alpha_{0}+x\alpha_{1}+\beta_{r}+x\gamma_{r}$$

or equivalently, y~x+race+x:race, with group-specific intercepts $\mu (0,r)=\alpha_{0}+\beta_{r}$ *and* group-specific slopes $\mu_{x}^{\prime }(r)=\alpha_{1}+\gamma_{r}$ for each race group r.

### Example 2 (Two-way ANOVA).

Suppose we have K=2 categorical variables race and sex with $L_{R}$ and $L_{S}$ groups, respectively. The main-only model (1) is then

(6) $$\mu^{M}(r,s)=\alpha_{0}^{M}+\beta_{1,r}^{M}+\beta_{2,s}^{M}$$

or equivalently, y~race+sex, while the cat-modified model (2) is

(7) $$\mu (r,s)=\alpha_{0}+\beta_{1,r}+\beta_{2,s}+\gamma_{rs}$$

or equivalently, y~race+sex+race:sex.

The central challenge is that expanding from the main-only model to the cat-modified model alters the interpretations, estimates, and inference for the *main effects*: the parameters $\left\{\alpha_{0}^{M},\alpha^{M},\beta_{k,c_{k}}^{M}\right\}$ in (1) or $\left\{\alpha_{0},\alpha ,\beta_{k,c_{k}}\right\}$ in (2). If these impacts are detrimental, then a quantitative modeler may be reluctant to include cat-modifiers. The key determinant is the model parameterization or *identification* strategy used for the categorical variable coefficients. Specifically, both models (1) and (2) require additional constraints to interpret and estimate the model parameters: the main-only intercepts $\left\{\alpha_{0}^{M},\beta_{k,c_{k}}^{M}\right\}$ are overparameterized, while the cat-modified intercepts $\left\{\alpha_{0},\beta_{k,c_{k}},\gamma_{k,k^{\prime },c_{k},c_{k^{\prime }}}\right\}$ and slopes $\left\{\alpha ,\gamma_{k,c_{k}}\right\}$ are overparameterized. The identifications determine the interpretations of all main and interaction parameters and the statistical properties of their estimators.

The most popular identification strategies are problematic for cat-modified models. *Reference group encoding* (RGE) is the overwhelming default, including for all major software implementations of linear regression (R, SAS, Python, MATLAB, Stata, etc.). With RGE, a reference group is selected for each categorical variable $C_{k}$ and removed: $\beta_{k,1}^{M}=0$ for all k in (1) and $\beta_{k,1}=0,\gamma_{k,1}=0,\gamma_{k,k^{\prime },1,c_{k^{\prime }}}=\gamma_{k,k^{\prime },c_{k},1}=0$ for all ($c_{k},c_{k^{\prime }}$) in (2) (using 1 for each reference group without loss of generality). This is equivalent to using $L_{k}-1$ “dummy variables” to encode each $C_{k}$. Despite the simplicity of RGE, the implied notion of “main effects” in (2) significantly impedes the use of cat-modifiers. For the main-only model, the jth main effect is a *global* slope, $\alpha_{j}^{M}=\mu_{x_{j}}^{M^{\prime }}$, invariant of c; yet for the cat-modified model, RGE fixes $\gamma_{j,k,1}=0$ for all j so that $\alpha_{j}=\mu_{x_{j}}^{\prime }(1)$ is the group-specific $x_{j}$-effect with *all* categorical variables set to their reference groups (c=1).

First, this main effect parameterization is statistically inefficient: SEs for ${\overset{ˆ}{\alpha }}_{j}$ are typically larger than those for ${\overset{ˆ}{\alpha }}_{j}^{M}$—the intersection of all reference groups is a subset of the data with a much smaller effective sample size—while the group-specific $x_{j}$-effect $\mu_{x_{j}}^{\prime }(c)$ may be smaller for the reference groups (c=1) than for other groups or globally (i.e., $\alpha_{j}^{M}$). We illustrate this effect in Table 1: with RGE, the main effects for the cat-modified model are attenuated and sacrifice power compared to those for the main-only model (see also Sections 4 and 5). Similar effects occur for categorical-categorical interactions (see Table E.1). Of course, these parameters refer to different functionals of μ(x,c); yet crucially, they are presented *identically* as “main effects” in statistical software output and manuscript tables (Knol et al. 2009). In fact, fewer than half of recent social science publications even reported the reference category (Johfre and Freese 2021). This leads to misleading conclusions about effect magnitudes, directions, and heterogeneity (Kowal 2024).

Second, RGE is inequitable: the main effect elevates a single group above the others. In Table 1, White is the reference group: the main effect $\alpha_{1}=\mu_{x}^{\prime }(\text{White})$ is the x-effect *for the White group*, while the interaction effect $\gamma_{r}=\mu_{x}^{\prime }(r)-\mu_{x}^{\prime }(\text{White})$ is the difference between the x-effect for race r and that for the White group. The choice of reference group also determines which estimates, SEs, and p-values are output by default (Table 1). RGE presents the reference groups (main effects) as “normal” and the other groups (interaction effects) as “deviations from normal”. This framing is known to bias the interpretations of results—often to the detriment of underrepresented groups (Chestnut and Markman 2018). The issues are compounded for regularized regression: when coefficient estimates are shrunk toward zero, the group-specific slopes are *statistically biased* toward the reference group slope ($\gamma_{r}\to 0\;\text{implies}\;\mu_{x}^{\prime }(r)\to \mu_{x}^{\prime }(\text{White})$). Beyond the obvious inequities—including (racial, gender, etc.) bias in the estimators—this shrinkage obscures potential differences between the x-effects for dominant (e.g., White, Male) and nondominant groups. These issues are explored for race in Kowal (2024), but also raise serious concerns for other protected groups (sex, national origin, religion, disability, etc.). Thus, RGE undermines progress toward statistical methods that promote equity (Chen et al. 2021).

Finally, RGE is difficult to interpret: each main effect and interaction in (2) must be traced back to *all* reference groups. Consider Example 2 (and Table E.1), using White and Male for the reference groups: the main effects in the cat-modified model (7) are $\alpha_{0}=\mu (\text{White},\text{Male})$, $\beta_{1,r}=\mu (r,\text{Male})-\mu (\text{White},\text{Male})$ for each race r, and $\beta_{2,s}=\mu (\text{White},s)-\mu (\text{White},\text{Male})$ for each sex s. Each main effect is anchored at both reference groups, which then affects the interpretations of the interaction effects $\gamma_{rs}$. This may be reasonable in some settings with a natural reference group (e.g., a control group), but RGE becomes increasingly difficult to interpret with multiple categorical covariates and interactions as in (2).

An alternative identification strategy uses *sum-to-zero* constraints (STZ). STZ identifies the parameters by restricting the group-specific coefficients via $\sum_{\ell =1}^{L_{k}}\beta_{k,\ell }^{M}=0$ for all k in the main-only model and $\sum_{\ell =1}^{L_{k}}\beta_{k,\ell }=0,\sum_{\ell =1}^{L_{k}}\gamma_{j,k,\ell }=0$ for j=1,…,p, and $\sum_{\ell =1}^{L_{k}}\gamma_{k,k^{\prime },\ell ,c_{k^{\prime }}}=0$ and $\sum_{\ell =1}^{L_{k}}\gamma_{k,k^{\prime },c_{k},\ell }=0$ for all ($c_{k},c_{k^{\prime }}$) in the cat-modified model. STZ is common for ANOVA models (Scheffe 1999; Fujikoshi 1993) and has been incorporated into regularized regression (Lim and Hastie 2015). STZ eliminates the need for a reference group, and thus resolves the inequities of RGE. However, STZ does not offer any special statistical properties for estimation of (1) or (2), nor does it establish a clear connection between the “main effects” in (1) and (2). As a result, it is difficult to interpret the parameters under STZ, while the addition of cat-modifiers may have unpredictable or detrimental effects on the main effect estimates and inferences (see Section 4).

To address these limitations, we advocate and analyze *abundance-based constraints* (ABCs) for identification and estimation with cat-modified models. Broadly, ABCs identify parameters using group abundances (see Section 2). ABCs are sufficiently general and may be combined with ordinary least squares (OLS), maximum likelihood, and modern regularized estimation techniques. The benefits are summarized by “EEI”:

1. **E****fficiency:** ABCs permit the inclusion of cat-modifiers (a) *without* altering the main effect OLS estimates and (b) either maintaining or *increasing* their statistical power, under reasonable conditions;
2. **E****quity:** ABCs do *not* require a reference group and thus eliminate the potential for introducing alarming inequities under default approaches (RGE); and
3. **I****nterpretability:** main effects are identified as group-averaged parameters, interaction effects are group-specific deviations from these group-averaged parameters, and both sets of parameters inherit meaningful notions of sparsity.

ABCs effectively remove the impediments to cat-modifiers, thus, facilitating richer regression analyses of heterogeneous effects. Of course, ABCs cannot guarantee that cat-modifiers will be practically or statistically significant, especially when the effective sample sizes for interactions are small. Rather, with ABCs, there is virtually nothing to lose by expanding from the main-only model (1) to the cat-modified model (2); yet the potential gains include greater statistical power for the main effects and discovery of heterogeneous effects.

We emphasize that ABCs, in various forms and by other names, have deep historical roots, but have lacked sufficient motivation to encourage widespread adoption. Scheffe (1999) and Fujikoshi (1993) considered identification strategies for ANOVA models based on arbitrary group-specific weights. Ultimately, both adopted STZ. Sweeney and Ulveling (1972) suggested ABCs for the simple ANCOVA (4) so that the estimated intercept would equal the sample mean (see also Theorem 1). However, there was no consideration of cat-modifiers or multiple covariates and no case made for any of EEI. Among nonlinear models, Park et al. (2021) and Park et al. (2023) used an ABC-like approach to *avoid* estimating main effects, instead focusing exclusively on interactions to optimize individual treatment rules. For the two-way ANOVA (7), Wang and Lin (2024) briefly mentioned ABCs only to dismiss them, claiming they “complicate the interpretation of the model parameters and make it difficult to fit the model…especially when other covariates are present.” Here, we forcefully argue the opposite, embodied by EEI—each of which applies with multiple covariates present.

Contrasts provide an alternative perspective on identification of (1) and (2): dummy coding, effects coding, and weighted effects coding (WEC) are respectively linked to RGE, STZ, and ABCs. Although WEC has garnered recent support (Grotenhuis et al. 2017a, 2017b), this work did not consider general cat-modifiers or any EEI. Instead, WEC has been mainly limited to two-way ANOVAs (6) or (7) and only advocated in restrictive settings with “certain types of unbalanced data that are missing not at random” (Brehm and Alday 2022) or “categories of different sizes, and if these differences are considered relevant” (Grotenhuis et al. 2017b). Our case is much broader and more direct: ABCs are ideal to identify coefficients on any categorical variables and cat-modifiers should be included in many, if not all linear models.

Lastly, we acknowledge additional perspectives on cat-modified models. A widely-used approach is *subgroup analysis*, which subsets the data into groups (for all combinations of c) and then fits separate regression models (e.g., Pocock et al. 2004). The appeal is that it estimates group-specific slopes without the complicated interpretations of the parameters in (2) under default approaches (RGE). However, subgroup analysis does not provide estimates or inference for the main effects, cannot incorporate regularization or borrow information across groups, and does not allow direct testing for interaction effects. ABCs offer the same (and more) benefits without any of these drawbacks. Related, Searle, Speed, and Milliken (1980) advocated for marginal means. These quantities, like group-specific slopes and fitted values, are identical for all (minimally sufficient) identifications under maximum likelihood estimation. Thus, it does not distinguish among identification strategies. However, the identification strategy remains key for (a) *parameter* interpretation, estimation, and inference and (b) regularized regression and variable selection.

The article is organized as follows. We introduce ABCs in Section 2 for parameter identification and statistical estimation. Our main results on the properties of estimation and inference with ABCs are in Section 3. Simulation studies are in Section 4 and a real data example is in Section 5. We conclude in Section 6. Supplementary material includes proofs of all results, details for generalized linear models, additional theoretical and simulation results, and supporting data analysis. An R package lmabc is available online with detailed documentation and examples at https://drkowal.github.io/lmabc/.

## 2. Identification, Estimation, and Inference with ABCs

The goal of ABCs is to enforce model identifiability while maintaining EEI. We first describe the model parameterization and interpretation, and then show how to compute regularized regression estimators and inference using linearly-constrained optimization. The main properties for estimation and inference are in Section 3.

### 2.1. Parameter Identification with ABCs

For motivation, consider (5) from Example 1: identifiability is obtained by constraining $\sum_{r=1}^{L_{R}}\pi_{r}\beta_{r}=0$ and $\sum_{r=1}^{L_{R}}\pi_{r}\gamma_{r}=0$ for some chosen nonnegative weights ${\left\{\pi_{r}\right\}}_{r=1}^{L_{R}}$. RGE sets $\pi_{1}=1$ and $\pi_{r}=0$ for r>0, while STZ sets all $\pi_{r}=1$. Equivalently, we can write each constraint as an average, $E_{\pi }\left(\beta_{R}\right)=0$ and $E_{\pi }\left(\gamma_{R}\right)=0$, where R is a categorical random variable with probabilities ${\left\{\pi_{r}\right\}}_{r=1}^{L_{R}}$. This perspective offers a path to interpret the model parameters under the identifying weights ${\left\{\pi_{r}\right\}}_{r=1}^{L_{R}}$; all parameters are still fixed and nonrandom. Now, we can identify the main x-effect as the *average* of the group-specific slopes: $\alpha_{1}=E_{\pi }\left(\alpha_{1}+\gamma_{R}\right)=E_{\pi }\left\{\mu_{x}^{\prime }(R)\right\}$. Then ABCs adopt a natural choice for ${\left\{\pi_{r}\right\}}_{r=1}^{L_{R}}$: the (population or sample) group abundances.

**Figure 1.**
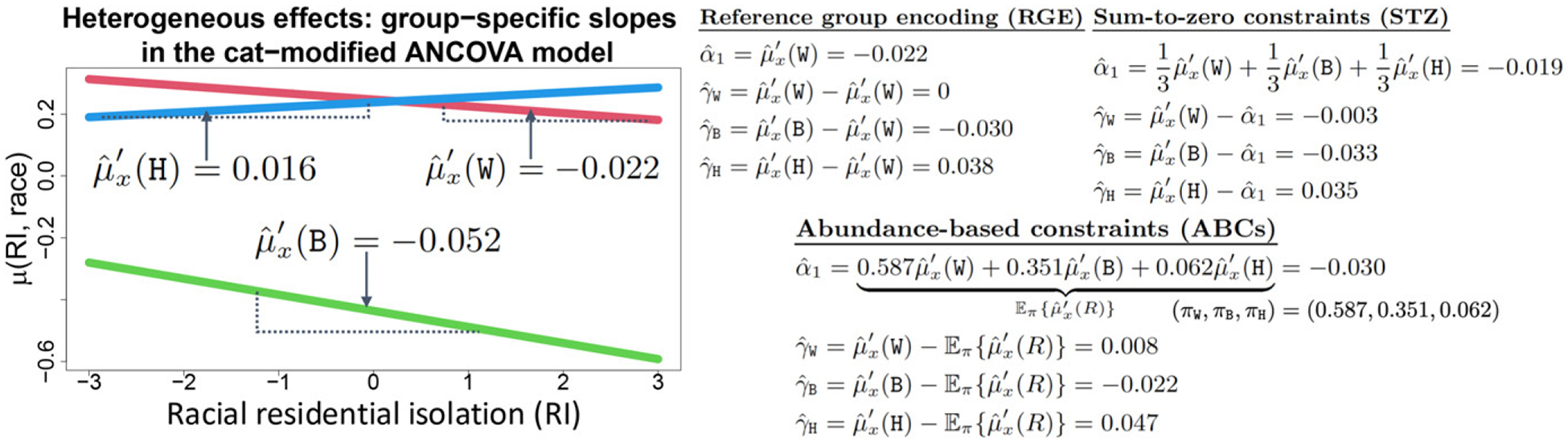
The connection between the estimated *group-specific slopes* $\left\{{\overset{ˆ}{\mu }}_{X}^{\prime }(r)={\overset{ˆ}{\alpha }}_{1}+{\overset{ˆ}{\gamma }}_{r}\right\}$ (left) and the estimated *model parameters* ${\overset{ˆ}{\alpha }}_{1},\left\{{\overset{ˆ}{\gamma }}_{r}\right\}$ (right) depends on the identification strategy. This example uses the same data as Table 1 (W = White, B = Black, H = Hispanic). For OLS estimation, the group-specific slope estimates are the same for RGE, STZ, and ABCs (not so for penalized estimation). However, the parameter estimates and interpretations are vastly different. RGE identifies parameters relative to the reference (W) slope; STZ uses an unweighted average; and ABCs use an average of the group-specific slopes based on the group abundances. With ABCs, the estimated RI main effect (${\overset{ˆ}{\alpha }}_{1}=-0.030$) most closely resembles the estimated effect from the main-only model (${\overset{ˆ}{\alpha }}_{1}^{M}=-0.036$); see Theorem 3.

For illustration, Figure 1 compares the model parameterizations under RGE, STZ, and ABCs using the same data as Table 1. In each case, the slope parameters $\alpha_{1}$ and ${\left\{\gamma_{r}\right\}}_{r=1}^{L_{R}}$ have a different link to the group-specific slopes $\mu_{x}^{\prime }(r)=\alpha_{1}+\gamma_{r}$, due to the differences in the implicit choices of weights ${\left\{\pi_{r}\right\}}_{r=1}^{L_{R}}$. Here, ABCs use the sample proportions, ($\left.{\overset{ˆ}{\pi }}_{\text{W}},{\overset{ˆ}{\pi }}_{\text{B}},{\overset{ˆ}{\pi }}_{\text{H}}\right)=(0.587,0.351,0.062)$ to identify the main x-effect as the average of the group-specific x-effects: ${\overset{ˆ}{\alpha }}_{1}=E_{\overset{ˆ}{\pi }}\left\{{\overset{ˆ}{\mu }}_{x}^{\prime }(R)\right\}={\overset{ˆ}{\pi }}_{\text{W}}{\overset{ˆ}{\mu }}_{x}^{\prime }(\text{W})+{\overset{ˆ}{\pi }}_{\text{B}}{\overset{ˆ}{\mu }}_{x}^{\prime }(\text{B})+{\overset{ˆ}{\pi }}_{\text{H}}{\overset{ˆ}{\mu }}_{x}^{\prime }(\text{H})=(0.587)(-0.022)+(0.351)(-0.052)+(0.062)(0.016)=-0.030$. Arguably, this a natural and interpretable parameterization for a global (main) x-effect.

More broadly, we define ABCs for the cat-modified model (2); special cases such as (1) simply omit the constraints for the omitted parameters. We express the ABCs in terms of $\overset{ˆ}{\pi }$, which is the joint proportions across all categorical variables $C={\left(C_{1},\ldots ,C_{K}\right)}^{⊤}$ in the data ${\left\{c_{i}\right\}}_{i=1}^{n}$. ABCs may be defined using population or sample proportions; we prefer the latter because they are always available and estimation properties are tractable and favorable (Section 3). First, ABCs for categorical main effects and categorical-continuous interactions are

(8a) $$E_{\overset{ˆ}{\pi }}\left(\beta_{k,C_{k}}\right)=0,\;k=1,\ldots ,K$$

(8b) $$E_{\overset{ˆ}{\pi }}\left(\gamma_{j,k,C_{k}}\right)=0,\;k=1,\ldots ,K,\;j=1,\ldots ,p.$$

Equivalently, ABCs may be expressed marginally and with summations: $\sum_{\ell =1}^{L_{k}}{\overset{ˆ}{\pi }}_{k,\ell }\beta_{k,\ell }=0$, where ${\left\{{\overset{ˆ}{\pi }}_{k,\ell }\right\}}_{\ell =1}^{L_{k}}$ are the sample proportions for each categorical variable $C_{k},k=1,\ldots ,K$, and then similarly for each ${\left\{\gamma_{j,k,\ell }\right\}}_{\ell =1}^{L_{k}}$. The key implication is that, while the cat-modified model (2) incorporates heterogeneity via mutual, group-specific slopes (3), ABCs concisely identify each main $x_{j}$-effect as the average of this group-specific slope:

(9) $$\alpha_{j}=E_{\overset{ˆ}{\pi }}\left\{\mu_{x_{j}}^{\prime }(C)\right\}.$$

ABCs parameterize each main $x_{j}$-effect by aggregating the group-specific slopes (3), each weighted by its respective abundance in the data. Unlike RGE, ABCs do not elevate any single (reference) group, and thus avoid the accompanying inequities.

The identification in (9) also guides interpretation of the group-specific slope parameters ${\left\{\gamma_{j,k,\ell }\right\}}_{\ell =1}^{L_{k}}$. Consider (5) from Example 1: $\gamma_{r}=\mu_{x}^{\prime }(r)-E_{\pi }\left\{\mu_{x}^{\prime }(R)\right\}$ is the difference between the group-specific slope for group r and the group-averaged slope. For the general cat-modified model (2), isolating $\gamma_{j,k,c_{k}}$ requires averaging over the remaining categorical variables $C_{-k}$ with joint proportions ${\overset{ˆ}{\pi }}_{-k}$ with $C_{k}=c_{k}$ fixed: $\gamma_{j,k,c_{k}}=E_{{\overset{ˆ}{\pi }}_{-k}}\left\{\mu_{x_{j}}^{\prime }\left(C_{-k},c_{k}\right)\right\}-E_{\overset{ˆ}{\pi }}\left\{\mu_{x_{j}}^{\prime }(C)\right\}$. Further simplifications are often available, since these averages only must include the categorical variables that act as cat-modifiers for $x_{j}$. In contrast with RGE, these group-specific coefficients are parameterized relative to a global main effect term (9), rather than a single (reference) group (White, Male, etc.).

For categorical-categorical interactions, ABCs identify $\left\{\gamma_{k,k^{\prime },c_{k},c_{k^{\prime }}}\right\}$ by requiring

(10) $$E_{{\overset{ˆ}{\pi }}_{C_{k}|C_{k^{\prime }}=\ell }}\left(\gamma_{k,k^{\prime },C_{k},\ell }\right)=0,\ell =1,\ldots ,L_{k^{\prime }}\;E_{{\overset{ˆ}{\pi }}_{C_{k^{\prime }}|C_{k}=\ell }}\left(\gamma_{k,k^{\prime },\ell ,C_{k^{\prime }}}\right)=0,\ell =1,\ldots ,L_{k}$$

for all ($C_{k},C_{k^{\prime }}$) interactions based on the *conditional* proportions for categorical variable $C_{k}$ given that the interacting variable $C_{k^{\prime }}$ belongs to group ℓ (and vice versa). We illustrate (10) using model (7) from Example 2: $E_{{\overset{ˆ}{\pi }}_{S|R=r}}\left(\gamma_{rS}\right)=0$ for $r=1,\ldots ,L_{R}$ and $E_{{\overset{ˆ}{\pi }}_{R|S=s}}\left(\gamma_{Rs}\right)=0$ for $s=1,\ldots ,L_{S}$, where ${\overset{ˆ}{\pi }}_{S|R=r}={\left\{{\overset{ˆ}{\pi }}_{rs}/\pi_{r}\right\}}_{s=1}^{L_{S}}$ is the conditional probability for each sex given race = r (similarly for ${\overset{ˆ}{\pi }}_{R|S=s}$). Equivalently, (10) may be expressed using the joint proportions $\overset{ˆ}{\pi }$: for Example 2, this is $\sum_{s=1}^{L_{s}}{\overset{ˆ}{\pi }}_{rs}\gamma_{rs}=0$ for all $r=1,\ldots ,L_{R}$ and $\sum_{r=1}^{L_{R}}{\overset{ˆ}{\pi }}_{rs}\gamma_{rs}=0$ for all $s=1,\ldots ,L_{S}$.

In conjunction, (8) and (10) constitute ABCs for the general cat-modified model (2). All ABCs can be written using expectations $E_{\overset{ˆ}{\pi }}(\cdot )$ under the *joint* probabilities $\overset{ˆ}{\pi }$ for the categorical variables C. We use these expectations to concisely identify and interpret model parameters; they do not change the general modeling context or assumptions for (2).

There are several compelling reasons to identify the categorical-categorical interactions with (10). First, it guarantees a global, group-averaged identification for the intercept:

*Lemma 1*. Under ABCs, the intercept parameter in (2) satisfies $E_{\overset{ˆ}{\pi }}\{\mu (0,C)\}=\alpha_{0}$.

ABCs produce clean expressions and simple interpretations for these main effects: while cat-modifiers induce group-specific intercepts μ(0,c) and group-specific slopes $\mu_{x_{j}}^{\prime }(c)$, ABCs identify global intercepts and global slopes by averaging over the groups, $\alpha_{0}=E_{\overset{ˆ}{\pi }}\{\mu (0,C)\}$ and $\alpha_{j}=E_{\overset{ˆ}{\pi }}\left\{\mu_{x_{j}}^{\prime }(C)\right\}$, respectively. This cannot occur for RGE and only occurs for STZ if the probabilities $\overset{ˆ}{\pi }$ are exactly uniform. Second, (10) orthogonalizes the main and interaction categorical effects: in fact, the OLS estimates of the main categorical effects $\left\{\beta_{k,c_{k}}\right\}$ are identical between models that do (7) or do not (6) include cat-modifiers (see Theorem 2 and its proof). Finally, (10) offers the interesting result that, if we were to instead combine the interacted covariates ($C_{k},C_{k^{\prime }}$) into a single categorical variable (e.g., race-sex) with $L_{k}L_{k^{\prime }}$ levels, the main effect ABCs (8) would be satisfied for this new categorical variable. Of course, doing so would sacrifice the ability to estimate the main effects $\left\{\beta_{k,c_{k}}\right\}$, but this internal consistency is reassuring.

For implementation, it is sufficient to enforce $L_{k}+L_{k^{\prime }}-1$ of the $L_{k}+L_{k^{\prime }}$ constraints in (10). The choice of omitted constraint is arbitrary, since all constraints (10) hold regardless:

*Lemma 2*. Suppose we apply (10) to all but one interaction term: $E_{{\overset{ˆ}{\pi }}_{C_{k}|C_{k^{\prime }}=\ell }}\left(\gamma_{k,k^{\prime },C_{k},\ell }\right)=0$ for $\ell =1,\ldots ,L_{k^{\prime }}$ and $E_{{\overset{ˆ}{\pi }}_{C_{k^{\prime }}|C_{k}=\ell }}\left(\gamma_{k,k^{\prime },\ell ,C_{k^{\prime }}}\right)=0$ for $\ell =2,\ldots ,L_{k}$. Then the same constraint holds for $\ell =1:E_{{\overset{ˆ}{\pi }}_{C_{k^{\prime }}|C_{k}=1}}\left(\gamma_{k,k^{\prime },1,C_{k^{\prime }}}\right)=0$.

Finally, we emphasize that ABCs (8) and (10) are designed for parameter identification in the general cat-modified model (2), which may be featured in generalized linear models (see the supplementary material, Section B), is readily generalizable for multivariate response regression, and includes many special cases, such as main-only models (1), ANCOVA models (Example 1), two-way ANOVA models (Example 2), etc.

### 2.2. Estimation, Inference, and Sparsity with ABCs

ABCs are linear constraints and thus readily compatible with regularized regression. First, we consolidate the cat-modified model (2) into a traditional regression structure: ${\left(\mu \left(x_{1},c_{1}\right),\ldots ,\mu \left(x_{n},c_{n}\right)\right)}^{⊤}=X\theta$, where X is the n×P matrix that includes an intercept, all (centered) continuous covariates, indicator variables for all levels of each categorical variable, and all specified interactions, and θ include all unknown regression coefficients. In the presence of at least one categorical covariate, X is rank deficient, say rank(X)=P-m. We represent all ABCs (8) and (10) generically as $A_{\overset{ˆ}{\pi }}\theta =0$, where is the m×P matrix of constraints with rank$\left(A_{\overset{ˆ}{\pi }}\right)=m$. Then, for a loss function ℒ(y,Xθ) for data $y={\left(y_{1},\ldots ,y_{n}\right)}^{⊤}$ and a coefficient penalty 𝒫(θ), we aim to solve

(11) $$\overset{ˆ}{\theta }(\lambda )=\text{arg}\;\underset{\theta }{\text{min}\;}ℒ(y,X\theta )+\lambda 𝒫(\theta )\;\text{subject to}\;A_{\overset{ˆ}{\pi }}\theta =0$$

and λ≥0 is a tuning parameter. We primarily focus on squared error loss $ℒ(y,X\theta )=\|y-X\theta {\|}^{2}$ and either unpenalized estimation (λ=0) or (group) lasso and ridge regression with λ selected by cross-validation. One way to solve (11) is to reparametrize to an unconstrained space with only P-m parameters. Let $A_{\overset{ˆ}{\pi }}^{⊤}=QR$ be the QR-decomposition with columnwise partitioning of the P×P orthogonal matrix $Q=\left(Q_{1:m}:Q_{\overset{ˆ}{\pi }}\right)$ and similarly, $R^{⊤}=\left(R_{1:m,1:m}:0\right)$. By construction, $A_{\overset{ˆ}{\pi }}Q_{\overset{ˆ}{\pi }}=0$, so that for any (P-m)-dimensional vector $\theta_{Q}$, the vector $\theta =Q_{\overset{ˆ}{\pi }}\theta_{Q}$ satisfies $A_{\overset{ˆ}{\pi }}\theta =0$. Then, letting $X_{Q}≔XQ_{\overset{ˆ}{\pi }}$, (11) is equivalently

(12) $$\overset{ˆ}{\theta }(\lambda )=Q_{\overset{ˆ}{\pi }}{\overset{ˆ}{\theta }}_{Q}\left(\lambda \right),\;{\overset{ˆ}{\theta }}_{Q}(\lambda )=\text{arg}\;\underset{\theta }{\text{min}\;}ℒ\left(y,X_{Q}\theta_{Q}\right)+\lambda 𝒫\left(Q_{\overset{ˆ}{\pi }}\theta_{Q}\right).$$

Regularized regression with ABCs simply requires (a) computing the QR decomposition of $A_{\overset{ˆ}{\pi }}^{⊤}$ and (b) solving an unconstrained regularized regression problem.

When ℒ is a negative log-likelihood, ${\overset{ˆ}{\theta }}_{Q}≔{\overset{ˆ}{\theta }}_{Q}(0)$ is a maximum likelihood estimator (MLE) and so is $\overset{ˆ}{\theta }$. Hence, usual properties for MLEs apply to estimators with ABCs. Under standard regularity conditions, (12) satisfies $\sqrt{n}(\overset{ˆ}{\theta }-\theta )\overset{d}{\to }N_{P}\left(0,Q_{\pi }ℐ{\left(\theta_{Q}\right)}^{-1}Q_{\pi }^{⊤}\right)$ where ℐ is the Fisher information associated with $\theta_{Q}$ and π is the joint population probabilities for the categorical covariates C. Thus, it is straightforward to construct confidence intervals and conduct hypothesis tests for the coefficients θ. When the model errors $y_{i}-\mu \left(x_{i},c_{i}\right)$ are Gaussian, uncorrelated, and homoscedastic, the OLS estimator under ABCs satisfies $\overset{ˆ}{\theta }\sim N_{P}\left\{\theta ,\sigma^{2}Q_{\overset{ˆ}{\pi }}{\left(X_{Q}^{⊤}X_{Q}\right)}^{-1}Q_{\overset{ˆ}{\pi }}^{⊤}\right\}$, even in finite samples. Although this distribution does not account for the sampling variability in $\overset{ˆ}{\pi }$, this is typically quite small relative to the variability in $\overset{ˆ}{\theta }$. Our empirical analyses suggest that no further adjustments are needed (see Section 4).

Finally, we emphasize the unique challenges of regularization and selection for cat-modified models. Selection of interaction effects has primarily focused on high-dimensional, continuous-continuous interactions (Bien, Taylor, and Tibshirani 2013). For cat-modified models with RGE, coefficient shrinkage introduces (racial, gender, etc.) biases: $\gamma_{j,k,c_{k}}\to 0\;\text{implies}\;\mu_{x_{j}}^{\prime }(c)\to \mu_{x_{j}}^{\prime }(1)$, so group-specific effects are pulled toward those for the reference (White, Male, etc.) groups. With ABCs, no such biases occur: $\gamma_{j,k,c_{k}}\to 0\;\text{implies}\;\mu_{x_{j}}^{\prime }(c)\to E_{\overset{ˆ}{\pi }}\left\{\mu_{x_{j}}^{\prime }(C)\right\}$ collapses to the group-averaged $x_{j}$-effect, which produces a reasonable notion of parameter sparsity.

When λ>0, it is possible to omit constraints and still obtain unique estimators. However, these estimators do not target identifiable parameters and thus are difficult to interpret. For lasso estimation, Kowal (2024) observes that such “overparametrized” estimation tends to reproduce RGE by implicitly selecting a reference group, and thus inherits the same limitations as RGE.

## 3. Theory for Estimation and Inference with ABCs

A central nuisance with interactions is that they change the main effect estimates and SEs. Here, we show that ABCs circumvent these challenges for cat-modifiers. The main point is that, with ABCs, the *addition* of cat-modifiers is either (a) harmless, since it has little to no impact on main effects estimates and inference, or (b) beneficial, since it can reveal heterogeneity and improve statistical power for the main effects. Our results make no assumptions about the true data-generating process for Y and do not apply for other identifications (RGE, STZ, etc.).

### 3.1. Estimation Invariance with ABCs

We establish conditions under which main effect OLS estimates are *invariant* to the addition of cat-modifiers under ABCs. First, consider OLS estimation of the intercept. For an enormous class of linear models—with arbitrarily many continuous covariates, categorical covariates, and categorical-categorical interactions—ABCs ensure that the OLS-estimated intercept is always *exactly* equal to the sample mean, ${\overset{ˆ}{\alpha }}_{0}=\overset{‾}{y}≔n^{-1}\sum_{i=1}^{n}y_{i}$.

*Theorem 1*. For *any* linear model of the form (2) with (a) centered continuous covariates ($\bar{x}=0$), (b) no categorical-continuous interactions (all $\gamma_{k,c_{k}}=0$), and (c) ABCs (8a) and (10), the OLS estimate of the intercept is ${\overset{ˆ}{\alpha }}_{0}=\overset{‾}{y}$.

Simple models such as y~race yield the same intercept estimate as more complex models like $\text{y}\sim {\text{x}}_{1}+\cdots +{\text{x}}_{p}+\text{race}+\text{sex}+\text{race}:\text{sex}$. This reaffirms the global interpretation of the intercept: under ABCs, ${\overset{ˆ}{\alpha }}_{0}$ targets the global intercept $\alpha_{0}=E_{\overset{ˆ}{\pi }}\{\mu (0,C)\}$ (Lemma 1). Of course, $\overset{‾}{y}$ is a good estimator for the marginal expectation of Y, so ${\overset{ˆ}{\alpha }}_{0}$ is appropriately global—even in the presence of categorical variables and their interactions. For models with at least one categorical variable, this result cannot occur for any other identification (RGE, STZ, etc.). Theorem 1 extends Sweeney and Ulveling (1972) to allow for categorical-categorical interactions and arbitrarily many continuous covariates.

Next, consider the impact of adding categorical-categorical interactions on estimation of the main effects. For concreteness, we consider a two-way ANOVA (Example 2).

*Theorem 2*. Under ABCs (8a) and (10), the OLS estimates of *all* main effects are identical under the main-only model (6) and the cat-modified model (7): ${\overset{ˆ}{\alpha }}_{0}^{M}={\overset{ˆ}{\alpha }}_{0},{\overset{ˆ}{\beta }}_{1,r}^{M}={\overset{ˆ}{\beta }}_{1,r}$ for all $r=1,\ldots ,L_{R}$, and ${\overset{ˆ}{\beta }}_{2,s}^{M}={\overset{ˆ}{\beta }}_{2,s}$ for all $s=1,\ldots ,L_{S}$.

This estimation invariance applies to *all* ($1+L_{R}+L_{S}$) main effects in (7). Implicitly, we assume that the OLS estimates exist and are unique (i.e., empty categories are not permitted), but otherwise there are no requirements on the data-generating process. In particular, there are no assumptions of independence or uncorrelateness between the categorical covariates and no assumptions about their relationship with Y. ABCs deliver a natural interpretation of the categorical variable coefficients: the main effects are deviations from the global mean (Theorem 1), while the interaction effects are deviations from the main effects in the main-only model (Theorem 2). This result validates our choice of ABCs (8) and especially (10). Again, such invariance does not occur for other identifications. We suitably generalize Theorem 2 in the supplementary material (Section C) to include additional continuous and categorical main effects as in (1).

Finally, we consider the addition of categorical-continuous interactions to main-only models. For clarity, we focus on a single categorical variable (K=1) with levels $r=1,\ldots ,L_{R}$, but showcase these principles empirically with multiple categorical variables (Section 5). Following Example 1, we begin with a single continuous covariate x(p=1). Let ${\overset{ˆ}{\sigma }}_{x[r]}^{2}≔n_{r}^{-1}s_{x[r]}^{2}-{\overset{‾}{x}}_{r}^{2}$ be the (scaled) sample variance of ${\left\{x_{i}\right\}}_{i=1}^{n}$ for each group r, where $n_{r}=n{\overset{ˆ}{\pi }}_{r},s_{x[r]}^{2}=\sum_{r_{i}=r}x_{i}^{2}$ and ${\overset{‾}{x}}_{r}=n_{r}^{-1}\sum_{r_{i}=r}x_{i}$. If the continuous covariate has the same scale for each group, then the OLS estimate of the coefficient on x is the same whether or not the cat-modifier is included.

*Theorem 3*. Under ABCs (8) and the equal-variance condition

(13) $${\overset{ˆ}{\sigma }}_{x[r]}^{2}={\overset{ˆ}{\sigma }}_{x[1]}^{2}\;\text{for all}\;r=1,\ldots ,L_{R},$$

the OLS estimates for the main-only model (4) and the cat-modified model (5) satisfy *estimation invariance*, ${\overset{ˆ}{\alpha }}_{1}^{M}={\overset{ˆ}{\alpha }}_{1}$.

We apply Theorem 3 as an approximation, ${\overset{ˆ}{\alpha }}_{1}\approx {\overset{ˆ}{\alpha }}_{1}^{M}$ whenever ${\overset{ˆ}{\sigma }}_{x[r]}^{2}\approx {\overset{ˆ}{\sigma }}_{x[1]}^{2}$ for all r, which is reasonably robust to deviations from (13) (see Table 1 and the supplementary material, Section D). This condition makes no requirements on the true associations between Y and (x,r) and generally allows the distribution of x to vary by r—as long as the scale is approximately constant. In particular, strong dependencies between x and r are permissible. Note that this result concerns categorical-continuous interactions only, so ABCs for categorical-categorical interactions (10) are not needed.

It is clarifying to consider violations of the equal-variance condition (13), so that x varies substantially for some groups, but varies little for others. This scenario does *not* invalidate estimation with ABCs; rather, it decouples the coefficients on x (i.e., the main x-effects) between the models that do (5) or do not (4) include a cat-modifier. Arguably, the main-only model (4) is no longer appropriate in this setting. The x-effect in (4) is $\alpha_{1}^{M}=\mu^{M}(x+1)-\mu^{M}(x)$, which considers a one-unit change in x *regardless of the group* r. But when (13) is violated, the scale of x—and a “one-unit change in x”—is no longer comparable across groups. Thus, *group-specific* slopes $\mu (x+1,r)-\mu (x,r)=\alpha_{1}+\gamma_{r}$ are appropriate, which mandates the cat-modified model. Instead of the global x-effect $\alpha_{1}^{M}$ from the main-only model, the cat-modified model with ABCs identifies a global x-effect via the group-averaged quantity $\alpha_{1}=E_{\overset{ˆ}{\pi }}\{\mu (x+1,R)-\mu (x,R)\}$ as in (9). In this setting, distinctness between $\alpha_{1}$ and $\alpha_{1}^{M}$ is appropriate. Finally, we do *not* require Theorem 3 (or the subsequent results) to use ABCs for estimation or inference. We contest that ABCs still provide more equitable and interpretable model output than other approaches.

This result can be extended for p continuous covariates, each of which is cat-modified: $\text{y}\sim {\text{x}}_{1}+\cdots +{\text{x}}_{p}+{\text{c}}_{1}+{\text{x}}_{1}:{\text{c}}_{1}+\cdots +{\text{x}}_{p}:{\text{c}}_{1}$. Here, the equal-variance condition (13) instead uses the (scaled) sample covariance between $x_{j}$ and $x_{h}$ in group $r,{\hat{\text{cov}}}_{r}\left(x_{j},x_{h}\right)≔n_{r}^{-1}\sum_{r_{i}=r}\left(x_{ij}-{\overset{‾}{x}}_{j}\right)\left(x_{ih}-{\overset{‾}{x}}_{h}\right)$.

*Theorem 4*. Consider the main-only model (1) and the cat-modified model (2), each with K=1 categorical variable. Under ABCs (8) and the equal-covariance condition ${\hat{\text{cov}}}_{r}\left(x_{j},x_{h}\right)={\hat{\text{cov}}}_{1}\left(x_{j},x_{h}\right)$ for all $r=1,\ldots ,L_{R}$ and each j,h=1,…,p, the OLS estimates satisfy $\overset{ˆ}{\alpha }={\overset{ˆ}{\alpha }}^{M}$.

Theorem 4 ensures estimation invariance for all p continuous main effects, each of which is cat-modified. This equalcovariance condition is stricter than (13). As with Theorem 3, we apply Theorem 4 as an approximation, so that $\overset{ˆ}{\alpha }\approx {\overset{ˆ}{\alpha }}^{M}$ when equal-covariance approximately holds.

Lastly, we establish a middle ground: $\text{y}\sim {\text{x}}_{1}+\cdots +{\text{x}}_{p}+{\text{c}}_{1}+{\text{x}}_{1}:{\text{c}}_{1}$, which is a cat-modified model with p continuous covariates and K=1 categorical variable, but now only $x_{1}$ is cat-modified. Instead of covariances between all pairs of covariates, the equal-variance condition now involves only $x_{1}$ and the residuals ${\overset{ˆ}{e}}_{1}$ from regressing $x_{1}$ on all other variables, ${\text{x}}_{1}\sim {\text{x}}_{2}+\cdots +{\text{x}}_{p}+{\text{c}}_{1}$.

*Theorem 5*. Consider the main-only model (1) with K=1 and the cat-modified model (2) with K=1 and interactions only with $x_{1}$ (fix $\gamma_{j,r}=0$ for all j>1 and $r=1,\ldots ,L_{R}$). Under ABCs (8) and the equal-variance condition ${\hat{\text{cov}}}_{r}\left(x_{1},{\overset{ˆ}{e}}_{1}\right)={\hat{\text{cov}}}_{1}\left(x_{1},{\overset{ˆ}{e}}_{1}\right)$ for all $r=1,\ldots ,L_{R}$, the OLS estimates satisfy ${\overset{ˆ}{\alpha }}_{1}^{M}={\overset{ˆ}{\alpha }}_{1}$.

To understand this modified equal-variance condition, we can equivalently express ${\hat{\text{cov}}}_{r}\left({\overset{ˆ}{e}}_{1},x_{1}\right)=n_{r}^{-1}\sum_{r_{i}=r}\left(x_{i1}^{2}-\right.\left.x_{i1}{\overset{ˆ}{x}}_{i1}\right)={\overset{ˆ}{\sigma }}_{x_{1}[r]}^{2}-{\hat{\text{cov}}}_{r}\left({\overset{ˆ}{x}}_{1},x_{1}\right)$, where ${\overset{ˆ}{x}}_{1}$ are the fitted values from ${\text{x}}_{1}\sim {\text{x}}_{2}+\cdots +{\text{x}}_{p}+{\text{c}}_{1}$. Theorem 5 requires that the variability in $x_{1}$ explained by the remaining (continuous and categorical) covariates is the same within each group. When this condition is violated, a one-unit change in $x_{1}$ holding *all else equal* among $x_{2},\ldots ,x_{p}$ is no longer comparable across groups r. As with Theorem 3, the main-only model x-effect $\alpha_{1}^{M}$ is no longer appropriate; group-specific $x_{1}$-effects $\mu_{x_{1}}^{\prime }(r)=\alpha_{1}+\gamma_{1,r}$ are preferred; and ABCs offer a substitute for the global slope parameter via the group-averaged $x_{1}$-effect, $\alpha_{1}=E_{\overset{ˆ}{\pi }}\left\{\mu_{x_{1}}^{\prime }(R)\right\}$.

For further context, we clarify the role of true (or population) parameters in main-only and cat-modified models. The parameters are identified by the chosen constraint (RGE, STZ, ABCs); estimates computed under the constraint target those identified parameters. A notable exception occurs for the continuous main effects $\alpha_{j}^{M}$ from the main-only model (1): these parameters are *not* anchored to any identification, $\alpha_{j}^{M}=\mu_{x_{j}}^{M^{\prime }}$, and their OLS estimates and inferences are identical under RGE, STZ, and ABCs (e.g., Table 1). For cat-modified models (2), no such agreement occurs—unless all cat-modifiers are extraneous, in which case the estimates ${\overset{ˆ}{\alpha }}_{j}$ all target $\alpha_{j}^{M}$. This underscores the appeal of ABCs afforded by Theorems 3–5: when cat-modifiers are extraneous, ABCs adopt the more efficient estimators ${\overset{ˆ}{\alpha }}_{j}^{M}$ from the main-only model that omits the extra terms. Otherwise, even when cat-modifiers are necessary, ABCs still target the identification-invariant main effects $\alpha_{j}^{M}$.

While Theorems 3–5 focus on estimation invariance for continuous main effects, similar properties emerge for categorical main effects. With continuous-categorical interactions, such estimation invariance is (approximately) enabled by the well-known approach of centering the continuous covariates ($\bar{x}=0$). However, unlike for continuous main effects (Theorems 3–5) or categorical main effects with categorical-categorical interactions (Theorem 2 and the supplementary material, Section C), this property is not unique to ABCs.

### 3.2. Powerful Inference with ABCs

A primary reason for the unpopularity of cat-modifiers is the loss of statistical power for the main effects. With RGE, cat-modifiers relegate the main effects to a single reference group, which shrinks the effective sample size and often attenuates global effects. Thus, quantitative modelers may be reluctant to include cat-modifiers for fear of larger p-values, wider confidence intervals, and less power to identify important effects. Consequently, potential race-, sex-, or other group-specific effects may remain hidden.

ABCs directly and uniquely address this challenge. With the addition of cat-modifier effects, we show that ABCs may actually *reduce* SEs for the main effects. The magnitude of this reduction increases with the effect size of the cat-modifier. Crucially, when the cat-modifier effect is unnecessary, the main effect SEs match, but do not inflate, those for a (correct) main-only model.

Consider two nested models, a main-only model and a cat-modified model. Our general result is that the cat-modified model with ABCs has smaller SEs for the main effects whenever the estimated *residual* variance is smaller for the cat-modified model,

(14) $${\overset{ˆ}{S}}^{2}\leq {\overset{ˆ}{S}}_{M}^{2}.$$

For the MLEs ${\overset{ˆ}{S}}^{2}=\|\overset{ˆ}{e}{\|}^{2}/n$ and ${\overset{ˆ}{S}}_{M}^{2}={\left\|{\overset{ˆ}{e}}_{M}\right\|}^{2}/n$, where $\overset{ˆ}{e}$ and ${\overset{ˆ}{e}}_{M}$ are the residuals from the cat-modified and main-only models, respectively, (14) is guaranteed: $\|\overset{ˆ}{e}{\|}^{2}\leq {\left\|{\overset{ˆ}{e}}_{M}\right\|}^{2}$, typically with strict inequality. More commonly, the unbiased estimators ${\overset{ˆ}{S}}^{2}=\|\overset{ˆ}{e}{\|}^{2}/\left(n-d_{M}-d\right)$ and ${\overset{ˆ}{S}}_{M}^{2}={\left\|{\overset{ˆ}{e}}_{M}\right\|}^{2}/\left(n-d_{M}\right)$ are used, where $d_{M}+d$ and $d_{M}$ are the number of identified parameters for the cat-modified and main-only models, respectively. In that case, (14) requires that the *adjusted*-$R^{2}$ for the cat-modified model exceeds that for the main-only model, or equivalently, (${\left\|{\overset{ˆ}{e}}_{M}\right\|}^{2}-\left.\|\overset{ˆ}{e}{\|}^{2}\right)/{\left\|{\overset{ˆ}{e}}_{M}\right\|}^{2}\geq d/\left(n-d_{M}\right)$, so that the (guaranteed) reduction in sum-squared-residuals from main-only to cat-modified must be large enough to justify the addition of d parameters. This requirement is modest: adjusted-$R^{2}$ is well-known to prefer overparametrized models, and thus (14) is likely to hold even when the cat-modifiers are extraneous (see Section 4.2). When cat-modifiers are indeed necessary, the reduction from ${\overset{ˆ}{S}}_{M}^{2}$ to ${\overset{ˆ}{S}}^{2}$ can be substantial.

We revisit each case from Section 3.1, beginning with a twoway ANOVA (Example 2).

*Theorem 6*. Under ABCs (8a) and (10) and (14), the OLS SEs of *all* main effects under the cat-modified model (7) are less than or equal to those under the main-only model (6): $\text{SE}\left({\overset{ˆ}{\alpha }}_{0}\right)\leq \text{SE}\left({\overset{ˆ}{\alpha }}_{0}^{M}\right),\text{SE}\left({\overset{ˆ}{\beta }}_{1,r}\right)\leq \text{SE}\left({\overset{ˆ}{\beta }}_{1,r}^{M}\right)$ for $r=1,\ldots ,L_{R}$, and $\text{SE}\left({\overset{ˆ}{\beta }}_{2,s}\right)\leq \text{SE}\left({\overset{ˆ}{\beta }}_{2,s}^{M}\right)$ for $s=1,\ldots ,L_{S}$.

Remarkably, Theorems 2 and 6 confirm that ABCs deliver the best possible result: adding cat-modifiers to the main-only model (6) does not change the main effect estimates, but potentially decreases their SEs. Thus, analysts may include cat-modifiers “for free”—with no negative consequences for the main effects—and gain the ability to infer possibly heterogeneous, group-specific effects. This result occurs precisely because ABCs ensure orthogonality between main and interaction effects—which notably does not occur for other (RGE, STZ) identifications.

A similar result applies for categorical-continuous interactions, again with the equal-variance condition:

*Theorem 7*. Under ABCs (8), equal-variance (13), and (14), the OLS SE for the main x-effect under the cat-modified model (5) is less than or equal to that under the main-only model (4): $\text{SE}\left({\overset{ˆ}{\alpha }}_{1}\right)\leq \text{SE}\left({\overset{ˆ}{\alpha }}_{1}^{M}\right)$. This result applies in the context of Theorem 3, but analogous extensions are available for Theorems 4 and 5; only the condition (14) must be added.

These results make minimal assumptions about the true datagenerating process and do *not* require independence or uncorrelatedness among the covariates. However, the OLS SEs are defined as usual, which implicitly refers to uncorrelated and homoskedastic error assumptions for both the main-only and cat-modified models. Thus, while Theorems 6 and 7 are direct statements about the SEs as statistics and do not require any assumptions on the error distributions, the utility of these results is clearly linked to these assumptions.

## 4. Simulations

### 4.1. Validating Invariance for Estimation and Inference

The first objective is to verify the theory of ABCs for estimation and inference invariance, focusing on the conditions in Section 3. We present results here for categorical-categorical interactions and consider categorical-continuous interactions in the supplementary material (Section D).

Given two categorical variables, say race and sex, what is the effect of including the race:sex interaction term on the estimates and SEs for the race and sex *main* effects? The theory of ABCs (Section 3) predicts that the estimates will be exactly the same, while the SEs may decrease if the interaction effect is sufficiently large. These results make no requirements on the data-generating process. Thus, we simulate data such that (a) race and sex are dependent, (b) the errors are non-Gaussian, and (c) ABCs are not satisfied.

Let race and sex be categorical variables with groups {A, B, C, D} and {uu, vv}, respectively; we use arbitrary labeling here to remain agnostic about particular race- or sex-specific effects in our synthetic data-generating process. For each of n=500 observations, we draw each race assignment with $\left(\pi_{a},\pi_{b},\pi_{c},\pi_{d})=(0.4,0.3,0.2,0.1\right)$, and then draw the sex assignment conditional on race with $\left(\pi_{uu},\pi_{vv}\right)\cdot |r=\text{A}=(0.4,0.6),{\left(\pi_{uu},\pi_{vv}\right)}_{\cdot |r=\text{B}}=(0.6,0.4),\left(\pi_{uu},\pi_{vv}\right)\cdot |r=\text{C}=(0.7,0.3)$, and $\left(\pi_{uu},\pi_{vv}\right)\cdot |r=\text{D}=(0.2,0.8)$. Thus, race and sex are dependent, and marginally $\pi_{uu}=\pi_{vv}=0.5$. The response variable y is simulated with expectation (7) with $\alpha_{0}=1,\beta_{c}=-1,\gamma_{b,vv}=\gamma$, and all other coefficients zero, or equivalently, μ(r,s)=1-I{r=C}+γI{r=B,s=vv} plus $t_{4}(0,1)$-distributed errors. Crucially, γ controls the magnitude of the race:sex effect: we consider γ=0 (no interaction effect), γ=0.5 (moderate interaction effect; see the supplementary material), and γ=1.5 (large interaction effect). We repeat this process to create 500 synthetic datasets.

For each simulated dataset, we fit the main-only model (6) and the cat-modified model (7) and compare the estimates and SEs for each main effect between the two models. These models are fit using ABCs, RGE (references r=A,s=uu), and STZ. This setting is favorable for RGE: the data-generating process satisfies RGE ($\beta_{a}=0,\beta_{uu}=0,\gamma_{a,uu}=0$), but not ABCs, and the reference groups are the most abundant groups for both race and sex. To aid comparisons, we omit the main effects from the reference groups, resulting in four main effects ($\beta_{b},\beta_{c},\beta_{d},\beta_{vv}$) to compare between the main-only and cat-modified models for ABCs, RGE, and STZ.

**Figure 2.**
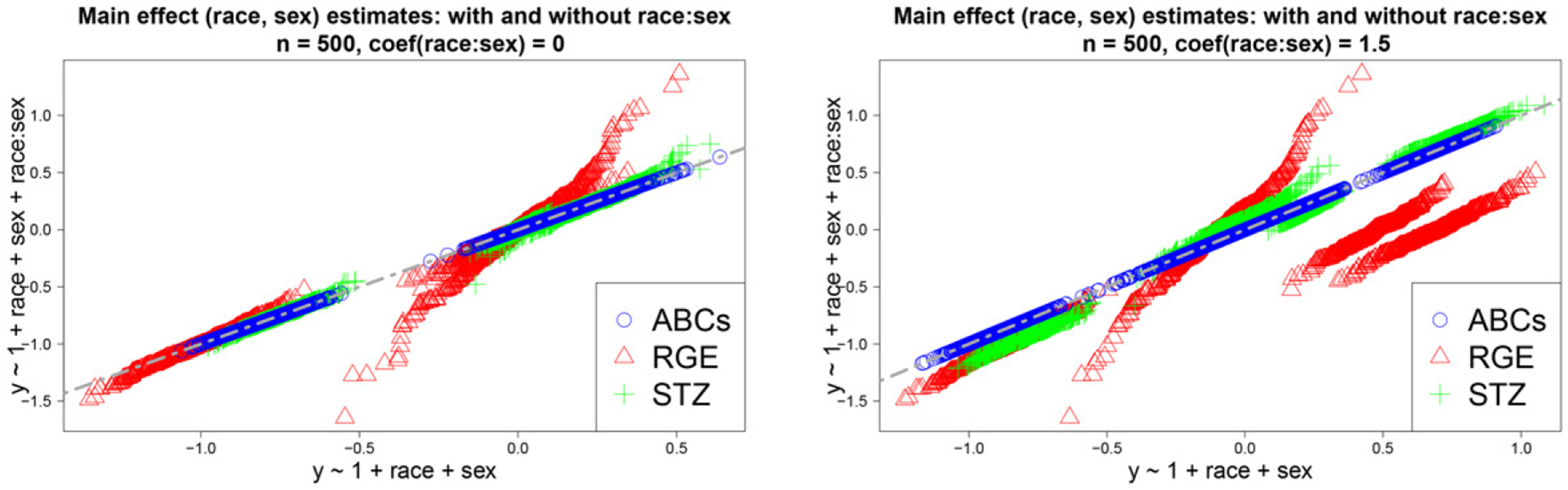
Estimates for all race and sex main effects for models that do (y-axis) and do not (x-axis) include the race:sex interaction across 500 simulated datasets. Under ABCs, all main effect estimates are *exactly* identical between the two models (45° line), regardless of whether the interaction effect is zero (γ=0, left) or large (γ=1.5, right). Such invariance does not hold for other identifications (RGE or STZ).

The estimates are in Figure 2. Under ABCs, *all* race and sex main effects are *exactly* identical between the models that do and do not include the race:sex interaction, confirming Theorem 2. This result persists regardless of the true data-generating process, including the magnitude of the interaction. No such invariance occurs for RGE or STZ: the inclusion of the interaction completely changes the estimates (and the interpretations) of the main effects.

**Figure 3.**
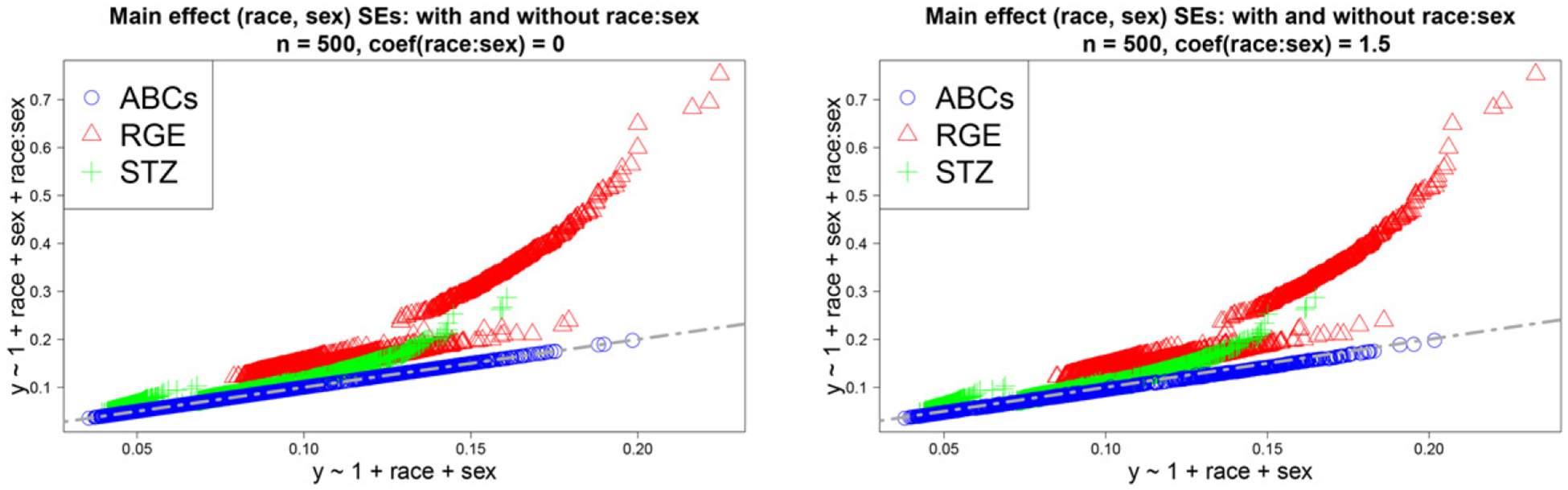
Standard errors (SEs) for all race and sex main effects for models that do (y-axis) and do not (x-axis) include the race:sex interaction across 500 simulated datasets. Under ABCs, the SEs are nearly identical between the two models (45° line) when the interaction effect is zero (γ=0, left) and slightly less (about a 5% reduction) for the cat-modified model when the interaction effect is larger (γ=1.5, right). The RGE and STZ main effect SEs increase substantially when the interaction term is included in the model (above 45° line) regardless of γ.

The SEs are in Figure 3. Under ABCs, the addition of the race:sex interaction has virtually no impact on the main effect SEs. For larger γ, the main effect SEs are slightly smaller (about a 5% reduction) for cat-modified model, as expected. More substantial SE reductions occur for larger interactions (γ≥5), although such large interaction effects are not usually expected in practice. Again, no such results occur for RGE: the SEs are much larger for the model that includes the race:sex interaction, regardless of γ.

These results must be interpreted carefully: the “main effects” under ABCs, RGE, or STZ target different functionals of μ(r,s). In fact, the OLS fitted values for $\overset{ˆ}{\mu }(r,s)$ are identical under each identification (this is not the case for regularized regression). However, each identification puts forth “main effects” in both the main-only and cat-modified model. We argue that the main effects under ABCs are superior: the estimates are *exactly* invariant to the inclusion of (race:sex) interactions and the SEs may decrease slightly. Uniquely, ABCs circumvent the traditional roadblocks to including interactions: the interpretations remain simple (and equitable) and there is no loss of statistical power for the main effects.

In the supplementary material (Section D), we revise this analysis for categorical-continuous interactions: given categorical race and continuous x, what is the effect of including the x:race interaction on the main x-effect? The theory of ABCs (Section 3) predicts that invariance for estimation and inference is contingent on the equal-variance condition (13). We investigate the sensitivity to this condition as well as to the magnitude of the interaction effect. Broadly, our findings resemble those from the categorical-categorical interaction case above—even with mild violations of the equal-variance condition (13), strong dependencies between race and x, and some model misspecification.

### 4.2. Evaluating Estimation and Inference with Cat-Modifiers

We evaluate the practical impacts of the estimation invariance and enhanced power of ABCs. The goal is to quantify the extent to which ABCs (a) maintain accurate estimates and precise uncertainty quantification when *extraneous* cat-modifiers are included and (b) improve estimation and reduce uncertainty when *necessary* cat-modifiers are included. The simulation design has three main features, described below.

First, we generate multiple, dependent categorical and continuous covariates. Dependent categorical variables race and sex are generated as in Section 4.1. Next, p=10 dependent continuous variables are generated as follows. We incorporate dependencies between race and $x_{j}$ for j=1,3,5,7,9 by drawing from the conditional distributions $\left[x_{j}|\right.\;\text{race}\;\left.=r\right]$ given by

(15) $$\left[x_{j}|\text{race}=r\right]\sim \left\{\begin{array}{ll} 5+N(0,1) & r=\text{A}\\ \sqrt{12}\;\text{Uniform}(0,1) & r=\text{B}\\ -5+t_{8}(0,1) & r=\text{C}\\ \text{Gamma}(1,1) & r=\text{D} \end{array}\right.$$

For each race group, $x_{j}$ has a unique distribution, with expectations that also vary by race. The remaining $x_{j}$ for j=2,4,6,8,10 are drawn independently from N(0,1). Some $x_{j}$ are highly correlated with race, which induces correlations among those x-variables with each other and with sex, while others are uncorrelated.

Second, the regression coefficients are constructed to satisfy both RGE and ABCs. These include an intercept $\alpha_{0}=1$, active main x-effects $\alpha_{j}=1$ for j=1,…,5, race main effects $\beta_{b}=1$ and $\beta_{c}=\beta_{d}=-1$, and cat-modifiers $\gamma_{b,j}=\gamma$ and $\gamma_{c,j}=\gamma_{d,j}=-\gamma$ for j=1,…,5; the remaining coefficients are all zero. RGE is enforced because all reference group coefficients are zero ($\beta_{a}=0,\beta_{uu}=0,\gamma_{a,uu}=0$, and $\gamma_{a,j}=0$ for all j=1,…,p), while ABCs are satisfied for the *population* proportions $\left(\pi_{a},\pi_{b},\pi_{c},\pi_{d}\right)=(0.4,0.3,0.2,0.1)$. Thus, it is meaningful to compare coefficient estimates and inference between RGE and ABCs because they target the same ground truth parameters (STZ is not satisfied and thus excluded). ABCs actually use the *sample* proportions and are at a slight disadvantage. All sex main and interaction effects are zero, since this is the only way to satisfy both RGE and ABCs for a variable with two groups.

Third, a parameter γ controls the magnitude of the cat-modifier (x:race) effect. We consider γ=0 for *extraneous* cat-modifiers and γ=1.5 for *necessary* cat-modifiers.

Using these covariates and coefficient values, the response variable y is simulated with expectation (2) plus Gaussian errors and a signal-to-noise ratio of one. We vary the sample size n∈{200,500,1000} and repeat this process to create 500 synthetic datasets.

For each synthetic dataset, we fit the main-only model $\text{y}\sim {\text{x}}_{1}+\cdots +{\text{x}}_{p}+\text{sex}+\text{race}$ and the cat-modified model $\text{Y}\sim \left({\text{x}}_{1}+\cdots +{\text{x}}_{p}+\right.\text{sex})*\text{race}$ that includes race interactions with all continuous covariates and sex, both under ABCs and RGE. The main-only model is favored when γ=0, while the opposite is true for γ>0. Either way, the true data-generating process is sparse, so both models include many extraneous parameters. The main-only model includes 15 identifiable parameters (9 true signals) while the cat-modified model includes 48 identifiable parameters. When γ=0, the cat-modified model estimates 33 extraneous (identified) parameters; even when γ>0, only 15 of those cat-modifier effects are nonzero.

Evaluation primarily focuses on the main x-effects ($\alpha_{1},\ldots ,\alpha_{10}$), which isolates the impacts of including extraneous (γ=0) or necessary (γ>0) cat-modifiers on estimation and inference for the main effects. For benchmarking, we also include evaluations for all (main and cat-modifier) coefficients. Note that for the main-only model, RGE and ABCs produce main x-effect estimates and SEs that are identical and nearly identical, respectively.

**Figure 4.**
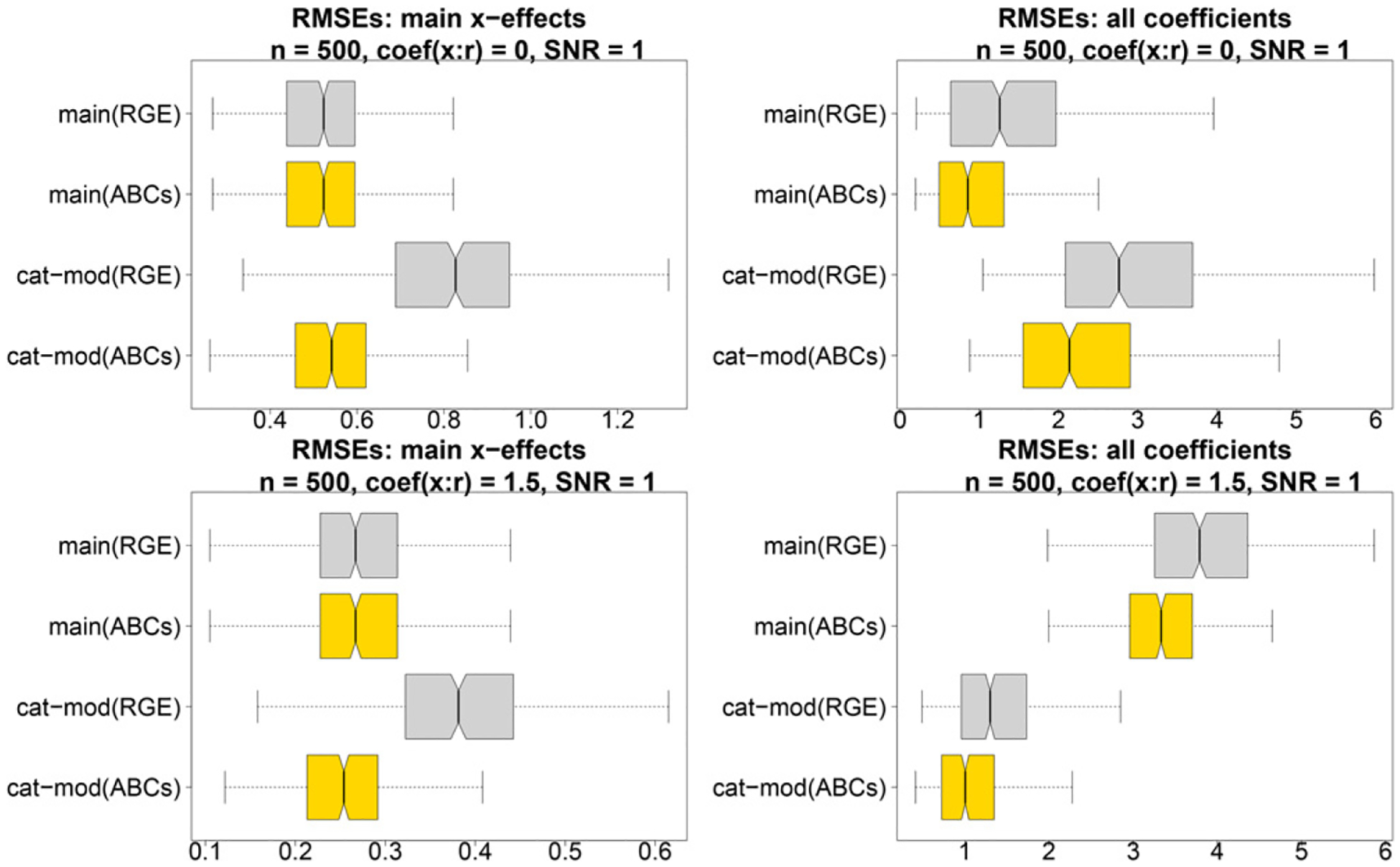
RMSEs for the main x-effects (left) and all coefficients (right) under main-only and cat-modified models with ABCs (gold) and RGE (gray). Boxplots are across 500 simulations; nonoverlapping notches indicate a difference in medians. Under ABCs, the cat-modified model main x-effect estimates are just as accurate as the main-only ones, even when the cat-modifiers are extraneous (top left), with slight gains when the cat-modifiers are necessary (bottom left). Neither result holds for RGE. For comparison, the accuracy across all coefficients is primarily determined by whether the correct model (main-only, top right; cat-modified, bottom right) is used.

Estimation accuracy is evaluated by root mean squared error (RMSE) for the regression coefficients (Figure 4). The cat-modified model *with ABCs* preserves estimation accuracy of the main x-effects, even when (all 33) cat-modifier effects are included unnecessarily (Figure 4, top left). When *some* cat-modifiers are necessary, the cat-modified model with ABCs delivers slightly more accurate main x-effect estimates than the main-only models (Figure 4, bottom left). Neither result holds for RGE. By comparison, estimation accuracy across all coefficients (Figure 4, right) overwhelmingly favors the correctly-specified model (main-only for γ=0, cat-modified for γ=1.5), regardless of RGE or ABCs. This result is not surprising, but rather serves as a contrast to emphasize the extraordinary robustness of *main* effect estimation accuracy for cat-modified models—but only under ABCs.

**Figure 5.**
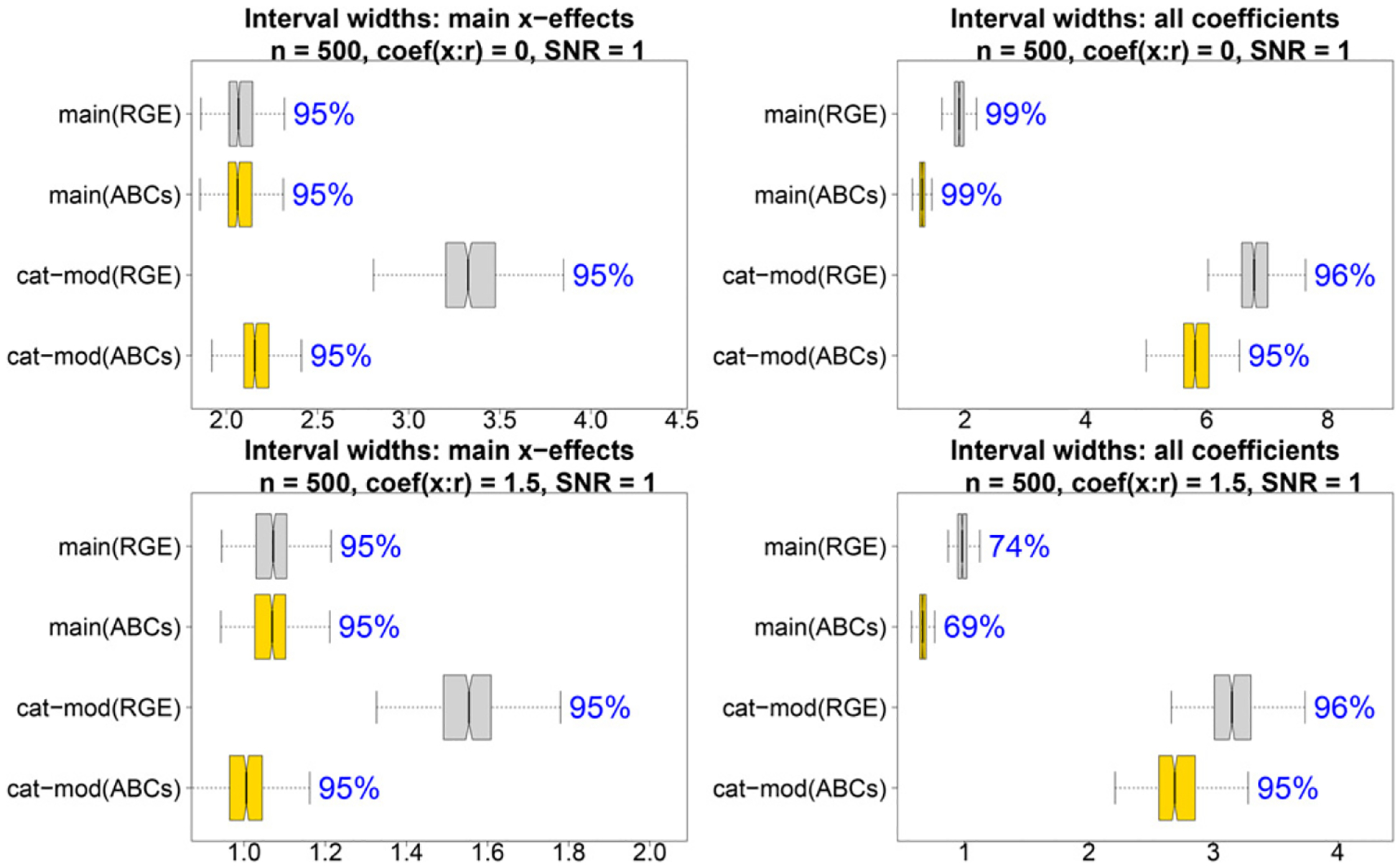
Interval widths (boxplots) and empirical coverage (blue annotations) for 95% confidence intervals for the main x-effects (left) and all coefficients (right) under main-only and cat-modified models with ABCs (gold) and RGE (gray). Under ABCs, inference for the main x-effects is nearly as powerful for the cat-modified model, even when the cat-modifiers are extraneous (top left), with greater power when the cat-modifiers are necessary (bottom left). Neither result holds for RGE. For comparison, extraneous cat-modifiers increase interval widths overall (top right), while the omission of necessary cat-modifiers sacrifices coverage for the main-only models (bottom right).

Inference is evaluated by mean interval widths and empirical coverage for 95% confidence intervals for the regression coefficients (Figure 5); narrow intervals are preferred, subject to nominal coverage. For ABCs, the cat-modified model offers nearly the same statistical power for the main x-effects as does the main-only model, even when (all 33) cat-modifier effects are included extraneously (Figure 5, top left). Compare that to inference for all coefficients (Figure 5, top right): here, the inclusion of extraneous cat-modifiers inflates interval widths by more than 300%. Clearly, this inferential robustness against extraneous cat-modifiers is a special property for (a) main effects and (b) ABCs. When *some* cat-modifiers are necessary, the cat-modified model with ABCs improves statistical power for the main x-effects compared to the main-only models. Again, no such results hold for RGE, for which the cat-modified model consistently sacrifices statistical power. Finally, as expected, main-only models fail to provide coverage for active cat-modified parameters (Figure 5, bottom right).

The supplementary material includes additional results for smaller (n=200) and larger (n=1000) sample sizes; predictive evaluations based on RMSEs for μ(x,c); and comparisons between ABCs and RGE for lasso and ridge regression, also including an “overparameterized” version that does not impose any constraints.

## 5. Application

We apply cat-modified regression to assess heterogeneity among factors linked to STEM educational outcomes. Our dataset^1^ links three administrative datasets to provide individual-level data for n=27,638 children in North Carolina (NC): NC Detailed Birth Records, NC Blood Lead Surveillance, and NC Standardized Testing Data; details are provided elsewhere (Initiative 2020; Kowal et al. 2021; Bravo et al. 2022). The STEM educational outcome variable $y_{i}$ is the end-of-4th-grade standardized math score for student i, centered and scaled by year of test (2010, 2011, or 2012). These math scores are linked with a rich collection of demographic, social, and environmental exposure variables. The continuous covariates are racial (residential) isolation (RI), which is a measure of structural racism based on neighborhood information; blood lead level (BLL), which measures lead exposure; birthweight percentile (BWTpct); mother’s age at time of child’s birth (mAge); and exposure to the air pollutant PM_2.5_ during the year prior to the exam (PM2.5). The continuous covariates are centered and scaled. The categorical covariates are mother’s race (race), child’s sex (sex), mother’s education level (mEdu), and an indicator of economically disadvantaged (EconDisadv) determined by participation in the National Lunch Program; see Table 2 for categorical levels and proportions.

Our linear regression analysis spans from main-only models to a variety of cat-modified models, expanding significantly upon the earlier models from Tables 1 and E.1. First, the *main-only* model includes each of these covariates (RI, BLL, BWTpct, mAge, PM2.5, race, sex, mEdu, and EconDisadv) but no interactions. The main-only model features a variety of interesting demographic, socio-economic, maternal, and environmental exposure variables, with 16 regression parameters (12 identified). Next, the *race-modified* model adds an interaction between race and every other covariate. This expansion allows for heterogeneous effects of each variable by race, thus providing insights into the myriad impacts of race on each child’s life course and educational outcomes, with 52 regression parameters (30 identified). Finally, the *cat-modified* model adds all pairwise categorical-continuous and categorical-categorical interactions. This instance of (2) allows the fullest (pairwise) extent of heterogeneous effects across the rich collection of demographic and socio-economic variables (race, sex, mEdu, and EconDisadv), with 103 regression parameters (55 identified). We fit each of these models under ABCs and RGE (references White, Male, lowest mEdu (mEdu<HS), and not EconDisadv).

**Figure 6.**
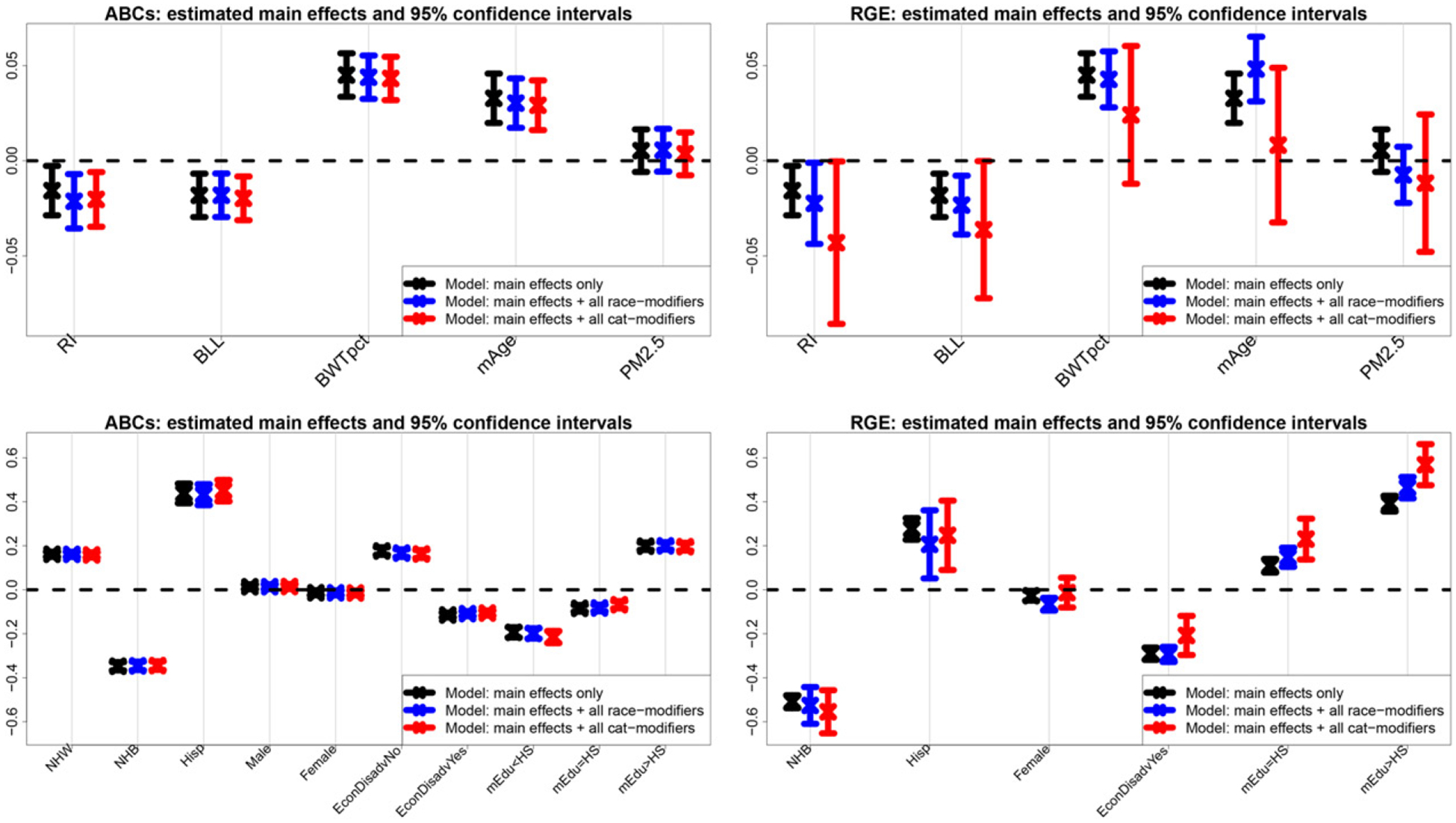
OLS estimates and 95% confidence intervals for continuous (top) and categorical (bottom) main effects under ABCs (left) and RGE (right) for three linear models: the *main-only model* (black) includes RI, BLL, BWTpct, mAge, PM2.5, race, sex, mEdu, and EconDisadv; the *race-modified* model (blue) adds interactions between race and every other covariate; and the *cat-modified* model (red) adds all pairwise categorical-continuous and categorical-categorical interactions. With ABCs, main effect inference is invariant to the cat-modifiers: all point and interval estimates are nearly identical across these substantially different models. With RGE, the main effect estimates shift and the intervals expand considerably as more cat-modifiers are added.

While each model offers potential for insight, a critical limitation of popular identification approaches, especially RGE, is that the estimates, inference, and interpretations of the main effects are highly sensitive to the choice of cat-modifiers. To see this, we present the main effect OLS estimates and 95% confidences intervals across these models in Figure 6. With RGE (right), the main effects shift and the intervals widen considerably—with increases from 160% to 230% in interval widths—upon adding race- (blue) and other (red) cat-modifiers. These main effects and accompanying interaction effects (not shown) are anchored at the reference groups and refer to different functionals of μ(x,c) under each model—even though the statistical output for the “main effects” is typically presented identically, regardless of any cat-modifiers. Thus, while cat-modified models are essential for heterogeneous effects, there is a cost incurred under RGE: each additional cat-modifier requires careful re-consideration of the main and interaction effects, which impedes statistical analysis and undermines interpretability.

The invariance of ABCs resolves these limitations: estimation and inference for the main effects (Figure 6, left) are nearly identical across these substantially different models. This occurs despite strong dependencies among the covariates (and interactions) with both continuous and categorical variables. ABCs effectively decouple the main effects from the cat-modifiers: even adding 87 parameters (43 identified) from the main-only model to obtain the cat-modified model does not lessen, and in some cases *increases* the statistical power for the main effects. With ABCs, the statistical analyst may consider these or other cat-modified models without compromising or complicating inferences for the main effects.

The full regression output from the cat-modified model with ABCs is in Tables 2 and E.3. Lower math scores are strongly (p<0.01) associated with racial (residential) isolation, lead exposure, lower (mother’s) education levels, and occur for non-Hispanic Black and economically disadvantaged students; higher math scores are strongly associated with birthweight percentile, mother’s age, and the opposing categories from above. ABCs provide output for all levels of all categorical variables, thus, eliminating the presentation bias of RGE that presents all output relative to the reference groups (White, Male, etc.). The regression output strongly supports heterogeneous effects, most notably via mother’s education level and with intersectionality of race and sex (e.g., Bauer 2014).

Finally, we simplify the heavily-parameterized cat-modified model by fitting a lasso regression under ABCs; λ is selected using 10-fold cross-validation and the one-standard-error rule (Hastie, Tibshirani, and Friedman 2009). The selected main effects (RI, BLL, BWTpct, mAge, race, mEdu, and EconDisadv) match the conclusions from Figure 6. Among interactions, coefficients from race:mEdu, race:mAge, race:EconDisadv, mEdu:mAge, and EconDisadv:mAge are selected. The accompanying coefficients of these cat-modifiers suggest that some positive effects are not as beneficial for minoritized groups: the positive effect of mother’s education (mEdu>HS) are attenuated for Black and Hispanic students, while the benefits of mother’s age are less so for lower mother’s education, Black, or economically disadvantaged students.

## 6. Conclusion

To encourage and enable statistical analysis of heterogeneous effects, we analyzed and advocated ABCs—an alternative parameterization and estimation strategy for cat-modified models that include categorical-continuous or categorical-categorical interactions. Unlike default methods, ABCs allow the inclusion of cat-modifiers “for free”: there is virtually no impact on the main effect estimates, while main effect inference is stable or more powerful. We rigorously proved these estimation and inference invariance properties and validated them empirically with extensive simulation studies. We also provided strategies for estimation and inference, including both generalized and regularized regression. Finally, we applied these tools to analyze STEM educational outcomes and showed how ABCs facilitate identification and estimation of (demographic) heterogeneous effects without incurring any costs—in estimation, inference, or interpretation—for the main effects.

Despite these many advantages, we note several caveats. First, ABCs may increase susceptibility to p-hacking. Because ABCs facilitate the inclusion of interactions, and with a large enumeration of potential interactions, there is a heightened potential for both discovery *and* false discovery. Proper statistical analyses require careful consideration of hypothesis tests with multiple testing corrections as appropriate. Second, ABCs cannot guarantee that cat-modifiers will be (practically or statistically) significant. Detection of heterogeneous effects often requires well-designed studies or large sample sizes. Finally, many categorical variables, especially race, sex, and other protected groups, are susceptible to misinterpetation, inaccurate labelings, and exclusions of small groups.

Promising avenues for future work include generalizations for additive models, Bayesian regression, and high-dimensional regression, where the invariance properties of ABCs may prove especially useful for both variable selection and efficient computing strategies. It is also possible to use ABCs for higher-order interactions. With one categorical term (e.g., categorical-continuous-continuous interactions) or two categorical terms (e.g., categorical-categorical-continuous interactions), we may immediately apply (8b) or (10), respectively. For higher-order categorical interactions, suitable generalizations of the conditional expectations in (10) may be developed.

## Supplementary Material

Supp 1

Supplementary material includes a PDF with proofs of all results, details for generalized linear models, additional theoretical and simulation results, and supporting data analysis; and R code files for reproducibility. An R package lmabc is available online with details and documentation at https://drkowal.github.io/lmabc/.

Supplementary materials for this article are available online. Please go to www.tandfonline.com/r/JASA.

## Figures and Tables

**Table 1.** Abbreviated output for the main-only model (4) and the cat-modified model (5) for North Carolina fourth grade reading scores y (see Section 5) with x = racial residential isolation (RI) and (mother’s) race.

Reference group encoding (RGE)				Abundance-based constraints (ABCs)			
Variable	Model	Estimate (SE)	*p*-value	Variable	Model	Estimate (SE)	*p*-value
RI	Main-only	−0.036 (0.007)	<0.001	RI	Main-only	−0.036 (0.007)	<0.001
	Cat-modified	−0.022 (0.011)	0.047		Cat-modified	−0.030 (0.007)	<0.001
RI:White	Cat-modified	ref	ref	RI:White	Cat-modified	0.008 (0.006)	0.157
RI:Black	Cat-modified	−0.030 (0.015)	0.036	RI:Black	Cat-modified	−0.022 (0.009)	0.014
RI:Hispanic	Cat-modified	0.038 (0.028)	0.163	RI:Hispanic	Cat-modified	0.047 (0.025)	0.059

NOTE: With RGE (left), the cat-modifier attenuates the RI main effect (red), inflates its SE, and suppresses race-specific RI effects. With ABCs (right), the RI main effect (blue) estimates and SEs are nearly invariant to the cat-modifier (see Section 3) and the output clearly shows that the RI effect is significantly negative and much worse for Black students.

**Table 2.** Cat-modified model output under ABCs for NC STEM education outcomes with all pairwise categorical-continuous and categorical-categorical interactions (see Table E.3 for EconDisadv effects).

Variable	Estimate (SE)	*p*-value
Intercept	−0.026 (0.008)	0.001
Racial isolation (RI)	−0.020 (0.007)	0.006
Blood lead level (BLL)	−0.020 (0.006)	0.001
Birthweight percentile (BWTpct)	0.043 (0.006)	<0.001
Mother’s age (mAge)	0.029 (0.007)	<0.001
PM_2.5_ exposure (PM2.5)	0.004 (0.006)	0.527
Mother’s race (race)		
White (58.7%)	0.158 (0.006)	<0.001
Black (35.1%)	−0.345 (0.010)	<0.001
Hispanic (6.2%)	0.451 (0.025)	<0.001
Child’s sex (sex)		
Male (49.9%)	0.015 (0.006)	0.010
Female (50.1%)	−0.015 (0.006)	0.010
Mother’s education level (mEdu)		
Did not complete high school (<HS; 24.0%)	−0.215 (0.014)	<0.001
Completed high school (=HS; 36.8%)	−0.068 (0.008)	<0.001
At least some postsecondary (>HS; 39.2%)	0.196 (0.009)	<0.001
White: Male	0.023 (0.006)	<0.001
Black:Male	−0.049 (0.010)	<0.001
Hisp:Male	0.056 (0.024)	0.019
White: Female	−0.023 (0.006)	<0.001
Black: Female	0.048 (0.009)	<0.001
Hisp:Female	−0.051 (0.022)	0.019
White:mEdu<HS	−0.042 (0.014)	0.003
Black:mEdu<HS	0.023 (0.017)	0.166
Hisp:mEdu<HS	0.062 (0.018)	0.001
White: mEdu=HS	0.000 (0.008)	0.971
Black: mEdu=HS	0.008 (0.011)	0.462
Hisp:mEdu=HS	−0.071 (0.038)	0.059
White: mEdu>HS	0.017 (0.007)	0.012
Black:mEdu>HS	−0.031 (0.016)	0.064
Hisp:mEdu>HS	−0.172 (0.066)	0.009
Male:mEdu<HS	−0.018 (0.012)	0.131
Female: mEdu<HS	0.017 (0.011)	0.131
Male:mEdu=HS	0.007 (0.008)	0.390
Female: mEdu=HS	−0.006 (0.007)	0.390
Male:mEdu>HS	0.005 (0.008)	0.570
Female: mEdu>HS	−0.005 (0.009)	0.570
RI:White	−0.002 (0.006)	0.795
RI:Black	−0.005 (0.009)	0.565
RI:Hisp	0.046 (0.025)	0.063
BLL:White	−0.003 (0.005)	0.582
BLL: Black	−0.004 (0.008)	0.620
BLL:Hisp	0.050 (0.023)	0.033
BWTpct:White	−0.002 (0.005)	0.731
BWTpct:Black	0.006 (0.008)	0.512
BWTpct:Hisp	−0.014 (0.023)	0.548
mAge:White	0.009 (0.006)	0.120
mAge: Black	−0.017 (0.009)	0.071
mAge: Hisp	0.009 (0.027)	0.733
PM2.5: White	−0.019 (0.005)	<0.001
PM2.5:Black	0.024 (0.008)	0.004
PM2.5:Hisp	0.037 (0.024)	0.123
RI:Male	0.001 (0.007)	0.835
RI:Female	−0.001 (0.007)	0.835
BLL:Male	0.002 (0.006)	0.793
BLL: Female	−0.002 (0.006)	0.793
BWTpct:Male	0.001 (0.006)	0.908
BWTpct:Female	−0.001 (0.006)	0.908
mAge:Male	−0.007 (0.007)	0.290
mAge: Female	0.007 (0.007)	0.290
PM2.5:Male	−0.005 (0.006)	0.370
PM2.5: Female	0.005 (0.006)	0.370
RI:mEdu<HS	−0.015 (0.012)	0.201
RI:mEdu=HS	−0.007 (0.009)	0.442
RI:mEdu>HS	0.016 (0.010)	0.104
BLL:mEdu<HS	−0.004 (0.011)	0.682
BLL:mEdu=HS	0.011 (0.008)	0.145
BLL:mEdu>HS	−0.008 (0.008)	0.350
BWTpct:mEdu<HS	−0.018 (0.011)	0.110
BWTpct:mEdu=HS	0.011 (0.008)	0.156
BWTpct:mEdu>HS	0.001 (0.008)	0.912
mAge:mEdu<HS	−0.039 (0.013)	0.003
mAge:mEdu=HS	−0.022 (0.009)	0.011
mAge:mEdu>HS	0.045 (0.009)	<0.001
PM2.5:mEdu<HS	−0.002 (0.011)	0.849
PM2.5:mEdu=HS	−0.013 (0.008)	0.091
PM2.5:mEdu>HS	0.013 (0.008)	0.096

NOTE: Categorical variable proportions are also indicated. Data are restricted to individuals with 37–42 weeks gestation, mAge ∈ [15,44] years, BLL ≤80μg/dL (and capped at 10μg/dL), birth order ≤ 4, no current English language learners, and residence in NC at the time of birth and time of fourth end-of-grade test.

## Footnotes

1 Data management, access, and analysis are governed by data use agreements and an Institutional Review Board–approved research protocol at the University of Illinois Chicago.
