# Supplementary material for: Facilitating Heterogeneous Effect Estimation via Statistically Efficient Categorical Modifiers
Source: J Am Stat Assoc. Author manuscript; Available in PMC 2026 Jun 11. (PMC13251731; doi:10.1080/01621459.2026.2635078)
Supplement: Supp 1 [file NIHMS2181855-supplement-Supp_1.zip › acc-form-JASA-TM-2024-0639R1.pdf]

Author Contributions Checklist Form

This form documents the artifacts associated with the article (i.e., the data and code supporting the computational findings) and describes how to reproduce the findings.

Part 1: Data

☐ This paper **does not** involve analysis of external data (i.e., no data are used or the only data are generated by the authors via simulation in their code).

☒ I certify that the author(s) of the manuscript have legitimate access to and permission to use the data used in this manuscript.

Abstract

There are three administrative datasets in North Carolina that are linked together to provide information about mother-child pairs:

1) Detailed birth records: mother's race, age, education level; child's sex, birthweight percentile for gestational age; racial residential isolation.
2) Blood lead surveillance: child's blood lead level.
3) Standardized testing data: 4th end-of-grade score, participation in National Lunch Program, one-year PM2.5 exposure.

Additional data documentation is available at https://www.cehidatahub.org.

Availability

☐ Data **are** publicly available
☒ Data **cannot be made** publicly available

If the data are publicly available, see the *Publicly available data* section. Otherwise, see the *Non-publicly available data* section, below.

Publicly available data

☐ Data are available online at:
☐ Data are available as part of the paper's supplementary material.
☐ Data are publicly available by request, following the process described here:

☐ Data are or will be made available through some other mechanism, described here:

Non-publicly available data

Discussion of lack of publicly available data:

The North Carolina dataset cannot be released due to privacy protections. However, access to the data can occur through establishing affiliation with the Children's Environmental Health Initiative (contact cehi@nd.edu). Additional data documentation is available at https://www.cehidatahub.org.

Description

File format(s)

☒ CSV or other plain text:
☐ Software-specific binary format (.Rda, Python pickle, etc.):
☐ Standardized binary format (e.g., netCDF, HDF5, etc.):
☐ Other (described here):

Data dictionary

☐ Provided by the authors in the following file(s):
☐ Data file(s) is (are) self-describing (e.g., netCDF files)
☒ Available at the following URL:

Data are documented in https://cehidatahub.org/hub/Cohort_2000 and described in https://doi.org/10.1073/pnas.2117868119 and https://doi.org/10.1002/sim.9099.

Additional information (optional)

Simulated data with similar characteristics (i.e., a mix of continuous and categorical variables with some dependencies) are generated in the accompanying R files. Additional examples are presented in the online documentation at https://drkowal.github.io/lmabc/.

Part 2: Code

Abstract

The code is based on the R package 'lmabc', available on GitHub and documented with examples at https://drkowal.github.io/lmabc/.

Description

Code format(s)

☒ Script files

  ☒ R ☐ Python ☐ Matlab

  ☐ Other:

☒ Package

  ☒ R ☐ Python ☐ MATLAB toolbox

  ☐ Other:

☐ Reproducible report

  ☐ R Markdown ☐ Jupyter notebook

  ☐ Other:

☐ Shell script

☐ Other (described here):

Supporting software requirements

Version of primary software used

R version 4.3.1

Libraries and dependencies used by the code

glmnet version 4.1-7 (optional; only for supplementary Figure D.7)
Matrix version 1.6-0 (optional; only for supplementary Figure D.7)

Supporting system/hardware requirements (optional)

Parallelization used

☒ No parallel code used
☐ Multi-core parallelization on a single machine/node
    Number of cores used:
☐ Multi-machine/multi-node parallelization
    Number of nodes and cores used:

License

☐ MIT License (default)
☐ BSD
☒ GPL v3.0
☐ Creative Commons
☐ Other (described here):

Additional information (optional)

Part 3: Reproducibility workflow

Scope

The provided workflow reproduces:
☐ Any numbers provided in text in the paper
☐ The computational method(s) presented in the paper (i.e., code is provided that implements the method(s))
☐ All tables and figures in the paper
☒ Selected tables and figures in the paper, as explained and justified here:

The workflow reproduces all simulation studies and corresponding figures from Section 4.1 (Figures 2-3), Section 4.2 (Figures 4-5), and the supplementary material (Figures D.1-D.7). The real data cannot be released and thus relevant output (Figures 1 and 6, Tables 1 and 2) cannot be reproduced exactly.

Workflow details

Format(s)

☐ Single master code file
☐ Wrapper (shell) script(s)
☐ Self-contained R Markdown file, Jupyter notebook, or other literate programming approach
☒ Text file (e.g., a readme-style file) that documents workflow
☐ Makefile
☐ Other (more detail in 'Instructions' below)

Instructions

The file ‘README.txt’ describes three R scripts corresponding to each simulation study. There are two main steps:

1) Installing ‘lmabc’, e.g., with pak::pak("drkowal/lmabc"))
2) Running the script that corresponds to the section to reproduce: ‘sims-4_1-cat-cat.R’ refers to Section 4.1, ‘sims-4_2-eval.R’ refers to Section 4.2, and ‘sims-AppD-cat-cts.R’ refers to the Appendix D.

Note that ‘helper_stz.R’ must be sourced in to run a competing method in ‘sims-4_1-cat-cat.R’ and ‘sims-AppD-cat-cts.R’.

Expected run-time

Approximate time needed to reproduce the analyses on a standard desktop machine:
☒ <1 minute
☐ 1-10 minutes
☐ 10-60 minutes
☐ 1-8 hours
☐ >8 hours
☐ Not feasible to run on a desktop machine, as described here:

Additional documentation (optional)

https://drkowal.github.io/lmabc/

Notes (optional)
