# Supplementary material for: Facilitating Heterogeneous Effect Estimation via Statistically Efficient Categorical Modifiers
Source: J Am Stat Assoc. Author manuscript; Available in PMC 2026 Jun 11. (PMC13251731; doi:10.1080/01621459.2026.2635078)
Supplement: Supp 1 [file NIHMS2181855-supplement-Supp_1.zip › supp.pdf]

Supplement to "Facilitating heterogeneous effect estimation via statistically efficient categorical modifiers"

Daniel R. Kowal

This supplementary file includes proofs of all results (Section A), details for generalized linear models (Section B), additional theoretical results (Section C), additional simulation results (Section D), and additional details and analyses of the North Carolina education data (Section E).

A Proofs

We first provide a sketch of the general proof technique. Our results require only basic linear algebra, but the notation can be cumbersome. Here, the goal is to provide clear intuition for our results and to put forth a blueprint to analyze similar invariance properties in other settings.

Consider two generic but nested models:

$\texttt{y} \sim \texttt{X}_* + \texttt{X}_0$

$\texttt{y} \sim \texttt{X}_* + \texttt{X}_0 + \texttt{X}_1$

The task is to establish conditions under which the OLS estimates of the coefficients on $\boldsymbol{X}_*$ are unchanged by the addition of $\boldsymbol{X}_1$, with $\boldsymbol{X}_0$ also present in both models. In our typical setting, $\boldsymbol{X}_*$ is a matrix of (continuous) covariates, $\boldsymbol{X}_0$ is a matrix of categorical indicator variables, and $\boldsymbol{X}_1$ contains cat-modifiers. Crucially, for *identifiable* estimation and inference, these matrices involving categorical covariates or cat-modifiers must already be parametrized to enforce the identifiable constraints, such as omitting certain columns for RGE or applying the QR reparametrization from Section 2.2 for ABCs.

The most relevant classical result is due to Frisch and Waugh (1933) and Lovell (1963):

Frisch-Waugh-Lovell (FWL) Theorem: For a partition of the $n \times p$ covariate matrix $\boldsymbol{X} = (\boldsymbol{X}_0 : \boldsymbol{X}_1)$ into p_0 and p_1 columns, the partition of the ordinary least squares estimator $\hat{\boldsymbol{\beta}} = (\hat{\boldsymbol{\beta}}_0^\top, \hat{\boldsymbol{\beta}}_1^\top)^\top$ satisfies $\hat{\boldsymbol{\beta}}_0 = (\boldsymbol{X}_0^\top \boldsymbol{E}_{01})^{-1} \boldsymbol{E}_{01}^\top \boldsymbol{y} = (\boldsymbol{E}_{01}^\top \boldsymbol{E}_{01})^{-1} \boldsymbol{E}_{01}^\top \boldsymbol{y}$, where $\boldsymbol{E}_{01} = (\boldsymbol{I}_n - \boldsymbol{H}_{X_1})\boldsymbol{X}_0$ is the $n \times p_0$ matrix of residuals from regressing each column of $\boldsymbol{X}_0$ on $\boldsymbol{X}_1$, $\boldsymbol{H}_{X_1} = \boldsymbol{X}_1(\boldsymbol{X}_1^\top \boldsymbol{X}_1)^{-1}\boldsymbol{X}_1^\top$ is the corresponding hat matrix for $\boldsymbol{X}_1$, and $\boldsymbol{y} = (y_1, \ldots, y_n)^\top$ is the vector of outcomes.

Applying the FWL Theorem, our target result occurs when $\texttt{residuals(X}_* \sim \texttt{X}_0\texttt{)} = \texttt{residuals(X}_* \sim \texttt{X}_0 + \texttt{X}_1\texttt{)}$, for which a sufficient condition is $\boldsymbol{X}_*^\top \boldsymbol{E}_{10} = 0$ where $\boldsymbol{E}_{10} =$

`residuals(X`$_1$ `~ X`$_0$`)`. More formally, let $\boldsymbol{H}_0 \coloneqq \boldsymbol{X}_0(\boldsymbol{X}_0^\top \boldsymbol{X}_0)^{-1}\boldsymbol{X}_0^\top$ be the hat matrix for the covariates $\boldsymbol{X}_0$ that are always included. Then the sufficient condition is

$$\boldsymbol{X}_*^\top(\boldsymbol{X}_1 - \boldsymbol{H}_0\boldsymbol{X}_1) = \mathbf{0} \tag{A.1}$$

or equivalently, $(\boldsymbol{X}_* - \boldsymbol{H}_0\boldsymbol{X}_*)^\top \boldsymbol{X}_1 = \mathbf{0}$, if we prefer to residualize $\boldsymbol{X}_*$ instead of $\boldsymbol{X}_1$. In the simpler case without a common $\boldsymbol{X}_0$ term, the requirement simplifies to $\boldsymbol{X}_*^\top \boldsymbol{X}_1 = \mathbf{0}$, where the role orthogonality is now abundantly clear.

In the presence of ABCs (or other linear constraints), we apply the reparametrization from Section 2.2 that replaces $\boldsymbol{X}_1$ with $\boldsymbol{X}_1\boldsymbol{Q}_{\hat{\boldsymbol{\pi}}}$ to enforce the constraints. The main condition (A.1) is now

$$\boldsymbol{X}_*^\top(\boldsymbol{X}_1 - \boldsymbol{H}_0\boldsymbol{X}_1)\boldsymbol{Q}_{\hat{\boldsymbol{\pi}}} = \mathbf{0}. \tag{A.2}$$

The key observation is that $\boldsymbol{A}_{\hat{\boldsymbol{\pi}}}\boldsymbol{Q}_{\hat{\boldsymbol{\pi}}} = \mathbf{0}$ by construction; this is true for the QR-based approach with *any* constraints of the form $\boldsymbol{A}_{\hat{\boldsymbol{\pi}}}\boldsymbol{\theta} = \mathbf{0}$, including but not limited to ABCs. Thus, the general requirement is to show that $\boldsymbol{X}_*^\top(\boldsymbol{X}_1 - \boldsymbol{H}_0\boldsymbol{X}_1)$ is row-wise proportional to $\boldsymbol{A}_{\hat{\boldsymbol{\pi}}}$, which produces the necessary zeros.

We apply this strategy for Theorems 3–7, but prove the main results in sequence.

Proof (Lemma 1). For simplicity, we prove this result for the case of (7), but the same ideas apply more generally. It is sufficient to show that $\mathbb{E}_{\hat{\boldsymbol{\pi}}}(\beta_{1,R} + \beta_{2,S} + \gamma_{RS}) = 0$. Direct application of (8a) implies $\mathbb{E}_{\hat{\boldsymbol{\pi}}}(\beta_{1,R} + \beta_{2,S} + \gamma_{RS}) = \mathbb{E}_{\hat{\boldsymbol{\pi}}_R}(\beta_{1,R}) + \mathbb{E}_{\hat{\boldsymbol{\pi}}_S}(\beta_{2,S}) + \mathbb{E}_{\hat{\boldsymbol{\pi}}}(\gamma_{RS}) = \mathbb{E}_{\hat{\boldsymbol{\pi}}}(\gamma_{RS})$, and further simplifying, $\mathbb{E}_{\hat{\boldsymbol{\pi}}}(\gamma_{RS}) = \sum_{r=1}^{L_R}\sum_{s=1}^{L_S} \hat{\pi}_{rs}\gamma_{rs} = 0$ since the internal summation is zero for all r by (10). □

Proof (Lemma 2). We prove this result for the case of (7) for simplicity. Applying (10) to all but $r = 1$, we have $\sum_{r=1}^{L_R} \hat{\pi}_{rs}\gamma_{rs} = 0$ for $s = 1, \ldots, L_S$ and thus $\gamma_{1s} = -\hat{\pi}_{1s}^{-1}\sum_{r=2}^{L_R}\hat{\pi}_{rs}\gamma_{rs}$. The conditional expectation is then $\mathbb{E}_{\hat{\boldsymbol{\pi}}_{S|R=1}}(\gamma_{RS}) = \sum_{s=1}^{L_S}\hat{\pi}_{1s}\gamma_{1s} = -\sum_{s=1}^{L_S}\sum_{r=2}^{L_R}\hat{\pi}_{rs}\gamma_{rs} = \sum_{r=2}^{L_R}\sum_{s=1}^{L_S}\hat{\pi}_{rs}\gamma_{rs} = 0$ since the internal summation equals zero for all $r > 1$. □

Proof (Theorem 1). Under OLS, $\bar{y}$ equals the sample mean of the fitted values $\{\hat{y}_i\}_{i=1}^n$; this is

true for ABCs, RGE, STZ, etc. Then we simplify:

$$
\begin{aligned}
\bar{y} = n^{-1}\sum_{i=1}^{n}\hat{y}_i &= n^{-1}\sum_{i=1}^{n}(\hat{\alpha}_0 + \boldsymbol{x}_i^\top\hat{\boldsymbol{\alpha}} + \sum_{k=1}^{K}\hat{\beta}_{k,c_k} + \sum_{k=1}^{K-1}\sum_{k'=k+1}^{K}\hat{\gamma}_{k,k',c_k,c_{k'}}) \\
&= \hat{\alpha}_0 + \bar{\boldsymbol{x}}^\top\hat{\boldsymbol{\alpha}} + \sum_{k=1}^{K}\sum_{c_k=1}^{L_k}\hat{\pi}_{k,c_k}\hat{\beta}_{k,c_k} + \sum_{k=1}^{K-1}\sum_{k'=k+1}^{K}\sum_{c_k=1}^{L_k}\sum_{c_{k'}=1}^{L_{k'}}\hat{\pi}_{k,k',c_k,c_{k'}}\hat{\gamma}_{k,k',c_k,c_{k'}} \\
&= \hat{\alpha}_0
\end{aligned}
$$

since the continuous covariates are centered ($\bar{\boldsymbol{x}} = \mathbf{0}$) and the main categorical effects and categorical-categorical interactions satisfy ABCs, so the interior summations equal zero for all k, k'. □

Proof (Theorem 2). Following the `race` and `sex` terminology from Example 2, define the design matrix by letting $\mathbf{1}$ be an n-dimensional vector of ones, $\boldsymbol{Z}_1$ the $n \times L_R$ matrix of `race` indicators with entries $[\boldsymbol{Z}_1]_{ir} = 1$ if $r_i = r$ and zero otherwise, and $\boldsymbol{Z}_2$ $n \times L_S$ matrix of `sex` indicators with entries $[\boldsymbol{Z}_2]_{is} = 1$ if $s_i = s$ and zero otherwise. Similarly, let $\boldsymbol{Z}_{12}$ be the $n \times L_R L_S$ matrix with indicators for the interaction terms. Consider the cross-produces of each main effect with the interaction matrix. First, $\mathbf{1}^\top\boldsymbol{Z}_{12}$ is the $1 \times L_R L_S$ matrix where each entry is the joint total by `race` and `sex`, i.e., $\sum_{i=1}^{n}\mathbb{I}(r_i = r, s_i = s)$ for each r, s combination. Next, $\boldsymbol{Z}_1^\top\boldsymbol{Z}_{12}$ is $L_R \times L_R L_S$, where each row r includes the totals $\sum_{i=1}^{n}\mathbb{I}(r_i = r, s_i = s)$ for all $s = 1, \ldots, L_S$ but zeros for columns with other `race` groups, $r' \neq r$. Similarly, $\boldsymbol{Z}_2^\top\boldsymbol{Z}_{12}$ is $L_S \times L_R L_S$, where each row s includes the totals $\sum_{i=1}^{n}\mathbb{I}(r_i = r, s_i = s)$ for all $r = 1, \ldots, L_R$ but zeros for columns with other `sex` groups, $s' \neq s$.

Estimation invariance occurs when these cross-products are zero. However, we must also account for identifiability constraints. Following Section 2.2, $\boldsymbol{Z}_{12}$ is replaced by $\boldsymbol{Z}_{12}\boldsymbol{Q}_{\hat{\boldsymbol{\pi}}}$, where $\boldsymbol{A}_{\hat{\boldsymbol{\pi}}}\boldsymbol{Q}_{\hat{\boldsymbol{\pi}}} = \mathbf{0}$ and $\boldsymbol{A}_{\hat{\boldsymbol{\pi}}}$ encodes the constraints on the interaction coefficients. Thus, it suffices to show that $\mathbf{1}^\top\boldsymbol{Z}_{12}\boldsymbol{Q}_{\hat{\boldsymbol{\pi}}} = \mathbf{0}$, $\boldsymbol{Z}_1^\top\boldsymbol{Z}_{12}\boldsymbol{Q}_{\hat{\boldsymbol{\pi}}} = \mathbf{0}$, and $\boldsymbol{Z}_2^\top\boldsymbol{Z}_{12}\boldsymbol{Q}_{\hat{\boldsymbol{\pi}}} = \mathbf{0}$, with each zero of the appropriate dimension. For ABCs, the latter two cross-products, when scaled by n^{-1}, exactly match the joint ABCs (10) in the form of $\boldsymbol{A}_{\hat{\boldsymbol{\pi}}}$, and thus are zero upon post-multiplication by $\boldsymbol{Q}_{\hat{\boldsymbol{\pi}}}$. Similarly, the first cross-product is also zero by applying the arguments from Lemma 1. □

Proof (Theorem 3). Let $\boldsymbol{y} = (y_1, \ldots, y_n)^\top$, $\boldsymbol{x} = (x_1, \ldots, x_n)^\top$, and $\boldsymbol{Z}$ be the matrix of categorical (`race`) indicators with entries $[\boldsymbol{Z}]_{ir} = 1$ if $r_i = r$ and zero otherwise. The cat-modifier

term is $\boldsymbol{Z}_X = \boldsymbol{D}_X \boldsymbol{Z}$ and $\boldsymbol{D}_X = \text{diag}(\boldsymbol{x})$. The goal is to show that, under the stated conditions, (A.2) holds with $\boldsymbol{x} = \boldsymbol{X}_*$, $\boldsymbol{X}_1 = \boldsymbol{Z}_X$, and $\boldsymbol{H}_0 = \boldsymbol{H}_Z = \boldsymbol{Z}(\boldsymbol{Z}^\top \boldsymbol{Z})^{-1}\boldsymbol{Z}^\top$ is the hat matrix for the categorical covariate.

For clarity, we provide more detailed results en route. Applying the FWL Theorem, the estimated coefficients under (4) satisfy $\hat{\alpha}_1^M = (\boldsymbol{x}^\top \hat{\boldsymbol{e}}_{x\sim r})^{-1}\hat{\boldsymbol{e}}_{x\sim r}^\top \boldsymbol{y}$, where $\hat{\boldsymbol{e}}_{x\sim r}$ is the vector of residuals from regressing the continuous variable $\{x\}_{i=1}^n$ on the categorical variable $\{r_i\}_{i=1}^n$ (i.e., $\boldsymbol{Z}$). Similarly, the estimated coefficients under (5) satisfy $\hat{\alpha}_1 = (\boldsymbol{x}^\top \hat{\boldsymbol{e}}_{x\sim r+Z_{XQ}})^{-1}\hat{\boldsymbol{e}}_{x\sim r+Z_{XQ}}^\top \boldsymbol{y}$, where $\hat{\boldsymbol{e}}_{x\sim r+Z_{XQ}}$ are the residuals from regressing the continuous variable $\{x\}_{i=1}^n$ on the categorical variable $\{r_i\}_{i=1}^n$ (i.e., $\boldsymbol{Z}$) *and* the reparametrized interaction term that enforces ABCs, $\boldsymbol{Z}_{XQ} = \boldsymbol{Z}_X \boldsymbol{Q}_{-(1:m)}$ (see Section 2.2). Thus, it suffices to show that $\hat{\boldsymbol{e}}_{x\sim r} = \hat{\boldsymbol{e}}_{x\sim r+Z_{XQ}}$, which occurs when the additional (interaction) coefficients from the latter model, say $\hat{\boldsymbol{b}}_{Z_{XQ}}$ (corresponding to $\boldsymbol{Z}_{XQ}$), are identically zero. Again using the FWL Theorem, these estimated coefficients are $\hat{\boldsymbol{b}}_{Z_{XQ}} = \boldsymbol{Q}_{-(1:m)}(\boldsymbol{Z}_{XQ}^\top \boldsymbol{E}_{Z_{XQ}})^{-1}\boldsymbol{E}_{Z_{XQ}}^\top \boldsymbol{x}$, where $\boldsymbol{E}_{Z_{XQ}}$ is the matrix of residuals from regressing $\boldsymbol{Z}_{XQ}$ on $\boldsymbol{Z}$, i.e., $\boldsymbol{E}_{Z_{XQ}} = \boldsymbol{Z}_{XQ} - \boldsymbol{H}_Z \boldsymbol{Z}_{XQ}$. Thus, showing $\boldsymbol{x}^\top \boldsymbol{E}_{Z_{XQ}} = \boldsymbol{0}$ is sufficient, and factoring $\boldsymbol{x}^\top \boldsymbol{E}_{Z_{XQ}} = (\boldsymbol{x}^\top \boldsymbol{Z}_X - \boldsymbol{x}^\top \boldsymbol{H}_Z \boldsymbol{Z}_X)\boldsymbol{Q}_{-(1:m)}$ shows the connection with (A.2).

First, observe that $\boldsymbol{x}^\top \boldsymbol{Z}_X = \boldsymbol{x}^\top \boldsymbol{D}_X \boldsymbol{Z} = (s_{x[1]}^2, \ldots, s_{x[L_R]}^2)$ is the vector of $s_{x[r]}^2 = \sum_{r_i = r} x_i^2$ across groups. Next, observe that $(\boldsymbol{Z}^\top \boldsymbol{Z})^{-1}\boldsymbol{Z}^\top \boldsymbol{Z}_X = \text{diag}(\{\bar{x}_r\}_r)$ contains the sample means of $\{x\}_{i=1}^n$ by each group r, and therefore $\boldsymbol{x}^\top \boldsymbol{H}_Z \boldsymbol{Z}_X = \boldsymbol{x}^\top \boldsymbol{Z}\text{diag}(\{\bar{x}_r\}_r) = (n_1 \bar{x}_1^2, \ldots, n_{L_R}\bar{x}_{L_R}^2)$ with $n_r = n\hat{\pi}_r$. Combining these results, we have $\boldsymbol{x}^\top \boldsymbol{E}_{Z_{XQ}} = \boldsymbol{v}^\top \boldsymbol{Q}_{-(1:m)}$, where $\boldsymbol{v}^\top = (s_1^2 - n_1\bar{x}_1^2, \ldots, s_{L_R}^2 - n_{L_R}\bar{x}_{L_R}^2) = n(\hat{\pi}_1 \hat{\sigma}_{x[1]}^2, \ldots, \hat{\pi}_{L_R}\hat{\sigma}_{x[L_R]}^2) = n\hat{\sigma}_{x[1]}^2 \hat{\boldsymbol{\pi}}^\top$ under the assumption that $\hat{\sigma}_{x[r]}^2 = \hat{\sigma}_{x[1]}^2$ is common for all r, which is precisely the equal-variance condition (13). Finally, the definition of $\boldsymbol{Q}_{-(1:m)}$ via ABCs implies that $\hat{\boldsymbol{\pi}}^\top \boldsymbol{Q}_{-(1:m)} = \boldsymbol{0}$, which proves the result. □

Proof (Theorem 4). Let $\boldsymbol{X}$ denote the $n \times p$ matrix of continuous covariates, $\boldsymbol{Z}$ the matrix of categorical dummy variables with entries $[\boldsymbol{Z}]_{ir} = 1$ if $r_i = r$ and zero otherwise, and $\boldsymbol{Z}_{XQ} = (\boldsymbol{Z}_{X_1 Q}, \ldots, \boldsymbol{Z}_{X_p Q})$ with $\boldsymbol{Z}_{X_j Q} = \boldsymbol{Z}_{X_j}\boldsymbol{Q}_{-(1:m)}$, $\boldsymbol{Z}_{X_j} = \boldsymbol{D}_{X_j}\boldsymbol{Z}$, and $\boldsymbol{D}_{X_j} = \text{diag}(\boldsymbol{x}_j)$. By the FWL Theorem, it suffices to show that $\boldsymbol{E}_M = \boldsymbol{E}$, where $\boldsymbol{E}_M = (\boldsymbol{I}_n - \boldsymbol{H}_Z)\boldsymbol{X}$ are the residuals from regressing each column of $\boldsymbol{X}$ on $\boldsymbol{Z}$ and $\boldsymbol{E}$ are similarly the residuals from regressing each column of $\boldsymbol{X}$ on $\boldsymbol{Z}$ *and* $\boldsymbol{Z}_{XQ}$. Thus, it is sufficient to show that the coefficients associated with $\boldsymbol{Z}_{XQ}$ in the latter regression are identically zero. Again using the FWL Theorem, we see that this occurs whenever $\boldsymbol{X}^\top \boldsymbol{E}_{Z_{XQ}} = \boldsymbol{0}$, where $\boldsymbol{E}_{Z_{XQ}} = \boldsymbol{Z}_{XQ} - \boldsymbol{H}_Z \boldsymbol{Z}_{XQ} = (\boldsymbol{Z}_{X_1 Q} -$

$\boldsymbol{H}_Z\boldsymbol{Z}_{X_1Q}, \ldots, \boldsymbol{Z}_{X_pQ}-\boldsymbol{H}_Z\boldsymbol{Z}_{X_pQ})$. Noticing that $\boldsymbol{X}^\top\boldsymbol{E}_{Z_{XQ}} = (\boldsymbol{X}^\top(\boldsymbol{Z}_{X_1Q}-\boldsymbol{H}_Z\boldsymbol{Z}_{X_1Q}), \ldots, \boldsymbol{X}^\top(\boldsymbol{Z}_{X_pQ}-\boldsymbol{H}_Z\boldsymbol{Z}_{X_pQ}))$, we consider the individual components $\boldsymbol{x}_h^\top(\boldsymbol{Z}_{X_jQ} - \boldsymbol{H}_Z\boldsymbol{Z}_{X_jQ}) = (\boldsymbol{x}_h^\top\boldsymbol{D}_{x_j}\boldsymbol{Z} - \boldsymbol{x}_h^\top\boldsymbol{H}_Z\boldsymbol{D}_{x_j}\boldsymbol{Z})\boldsymbol{Q}_{-(1:m)}$, each of which must equal the zero vector with dimension equal to the number of categories. Noting that $\boldsymbol{x}_h^\top\boldsymbol{D}_{x_j}\boldsymbol{Z} = (\ldots, s_r(j,h), \ldots)$ with $s_r(j,h) = \sum_{r_i=r} x_{ij}x_{ih}$ and $\boldsymbol{x}_h^\top\boldsymbol{H}_Z\boldsymbol{D}_{x_j}\boldsymbol{Z} = (\ldots, n_r\bar{x}_r(j)\bar{x}_r(h), \ldots)$ with $\bar{x}_r(j) = n_r^{-1}\sum_{r_i=r} x_{ij}$, we apply the same arguments as in Theorem 3. □

Proof (Theorem 5). Let $\boldsymbol{X}_0$ be the matrix of covariates that includes $\boldsymbol{X}_{-1}$ (i.e., all covariates but $\boldsymbol{x}_1$) and the categorical (race) indicators $\boldsymbol{Z}$ with entries $[\boldsymbol{Z}]_{ir} = 1$ if $r_i = r$ and zero otherwise, and let $\boldsymbol{H}_0 := \boldsymbol{X}_0(\boldsymbol{X}_0^\top\boldsymbol{X}_0)^{-1}\boldsymbol{X}_0^\top$ be its hat matrix. For the interaction terms, let $\boldsymbol{Z}_{x_1Q} = \boldsymbol{Z}_{x_1}\boldsymbol{Q}_{\hat{\boldsymbol{\pi}}}$ where $\boldsymbol{Z}_{x_1} = \boldsymbol{D}_{x_1}\boldsymbol{Z}$, $\boldsymbol{D}_{x_1} = \text{diag}(\boldsymbol{x}_1)$, and $\hat{\boldsymbol{\pi}}^\top\boldsymbol{Q}_{\hat{\boldsymbol{\pi}}} = \boldsymbol{0}$ enforces the ABCs for the interaction terms (see Section 2.2). Now, it is sufficient to show that $(\boldsymbol{x}_1^\top\boldsymbol{Z}_{x_1} - \boldsymbol{x}_1^\top\boldsymbol{H}_0\boldsymbol{Z}_{x_1})\boldsymbol{Q}_{\hat{\boldsymbol{\pi}}} = \boldsymbol{0}$ as in (A.2). First, observe that $\boldsymbol{x}_1^\top\boldsymbol{Z}_{x_1} = (\cdots s^2_{x1[r]} \cdots)$. Second, $\boldsymbol{x}_1^\top\boldsymbol{H}_0\boldsymbol{Z}_{x_1} = \hat{\boldsymbol{x}}_1^\top\boldsymbol{D}_{x_1}\boldsymbol{Z} = (\cdots \sum_{r_i=r}\hat{x}_{i1}x_{i1}\cdots)$ where $\hat{\boldsymbol{x}}_1 = \boldsymbol{H}_0\boldsymbol{x}$. Combining these results, we see that $\boldsymbol{v}^\top := \boldsymbol{x}_1^\top\boldsymbol{Z}_{x_1} - \boldsymbol{x}_1^\top\boldsymbol{H}_0\boldsymbol{Z}_{x_1} = (\cdots\sum_{r_i=r}(x_{i1}^2 - x_{i1}\hat{x}_{i1})\cdots)$. Consider the interior terms for each r: $\sum_{r_i=r}(x_{i1}^2 - x_{i1}\hat{x}_{i1}) = \sum_{r_i=r} x_{i1}\hat{e}_{i1} = n_r\widehat{\text{Cov}}_r(\hat{\boldsymbol{e}}_1, \boldsymbol{x}_1)$, where the latter equality holds because $\sum_{r_i=r}\hat{e}_{i1} = 0$ for each r due to the inclusion of $\boldsymbol{Z}$. Thus, $\boldsymbol{v}^\top = (\cdots n_r\widehat{\text{Cov}}_r(\hat{\boldsymbol{e}}_1, \boldsymbol{x}_1)\cdots) = k(\cdots n_r \cdots)$ for some constant k that does not depend on r, which implies that $\boldsymbol{v}^\top\boldsymbol{Q}_{\hat{\boldsymbol{\pi}}} = nk\hat{\boldsymbol{\pi}}^\top\boldsymbol{Q}_{\hat{\boldsymbol{\pi}}} = \boldsymbol{0}$ under ABCs. □

Proof (Theorem 6). The proof of Theorem 2 establishes orthogonality of ABCs-constrained interaction to the main effects under the same conditions. Given this orthogonality, the remainder of the proof follows the proof of Theorem 7 and is omitted for brevity. □

Proof (Theorem 7). Applying the same arguments from the proof of Theorem 3, the variances satisfy $\text{Var}(\hat{\alpha}_1^M) = \sigma_M^2(\boldsymbol{x}^\top\hat{\boldsymbol{e}}_{x\sim r})^{-1}$ and $\text{Var}(\hat{\alpha}_1) = \sigma^2(\boldsymbol{x}^\top\hat{\boldsymbol{e}}_{x\sim r+Z_{XQ}})^{-1}$, where σ_M^2 is the error variance from the main-only model and σ^2 is the error variance from the cat-modified model, assuming uncorrelated and homoskedastic errors in both models. These error assumptions are *not* required to prove the result, but do motivate the definition of the SE. Under the equal-variance condition (13), we previously showed that $\hat{\boldsymbol{e}}_{x\sim r} = \hat{\boldsymbol{e}}_{x\sim r+Z_{XQ}}$. Thus, the only difference in the variances of the estimators occurs because of the error variances, i.e., $\text{Var}(\hat{\alpha}_1)/\text{Var}(\hat{\alpha}_1^M) = \sigma^2/\sigma_M^2$. The SEs substitute point estimates for σ_M and σ: $\text{SE}(\hat{\alpha}_1^M) = \hat{S}_M\sqrt{(\boldsymbol{x}^\top\hat{\boldsymbol{e}}_{x\sim r})^{-1}}$ and

similarly,

$$\begin{aligned}
\mathrm{SE}(\hat{\alpha}_1) &= \hat{S}\sqrt{(\boldsymbol{x}^\top \hat{\boldsymbol{e}}_{x\sim r+Z_{XQ}})^{-1}} \\
&= \hat{S}\sqrt{(\boldsymbol{x}^\top \hat{\boldsymbol{e}}_{x\sim r})^{-1}} \\
&= \frac{\hat{S}}{\hat{S}_M}\mathrm{SE}(\hat{\alpha}_1^M) \\
&\leq \mathrm{SE}(\hat{\alpha}_1^M)
\end{aligned}$$

since $\hat{S} \leq \hat{S}_M$ under (14). □

B Generalized linear models with ABCs

Generalized linear models (GLMs) are immensely useful for regression analysis with a variety of data types, including continuous, count, binary, and categorical data. Broadly, GLMs require a choice of data distribution (e.g., Gaussian, Poisson, Bernoulli, etc.) and a link function g that replaces the expectation of Y, say $\mu(\boldsymbol{x}, \boldsymbol{c})$ with a transformed version, say $g\{\mu(\boldsymbol{x}, \boldsymbol{c})\}$, in the cat-modified model (2) (similarly for the main-only model (1)). With categorical covariates and cat-modifiers, identification constraints are needed for the regression coefficients exactly as in the ordinary (untransformed) linear model. ABCs again provide a suitable identification strategy, with straightforward estimation and inference: the loss function $\mathcal{L}$ in Section 2.2 is specified to incorporate the appropriate negative log-likelihood and link function.

More subtly, the presence of the link function g implies that interpretations of the coefficients will be different from those in the ordinary linear model (Section 2). For the main x_j-effects, recall that $\alpha_j = \mathbb{E}_{\hat{\boldsymbol{\pi}}}(\alpha_j + \sum_{k=1}^K \gamma_{j,k,C_k})$ under ABCs (8b), regardless of the data distribution or the link function. When the link g is *not* the identity, it is no longer the case that the internal quantity in the expectation equals $\mu'_{x_j}(\boldsymbol{C})$, and thus the previous interpretation from (9) requires modifications. Most generally, cat-modified GLMs satisfy

$$g\{\mu(x_j + 1, \boldsymbol{x}_{-j}, \boldsymbol{c})\} - g\{\mu(x_j, \boldsymbol{x}_{-j}, \boldsymbol{c})\} = \alpha_j + \sum_{k=1}^K \gamma_{j,k,c_k}, \tag{B.1}$$

so under ABCs (8b) the main x_j-effect is

$$\alpha_j = \mathbb{E}_{\hat{\boldsymbol{\pi}}}[g\{\mu(x_j + 1, \boldsymbol{x}_{-j}, \boldsymbol{C})\} - g\{\mu(x_j, \boldsymbol{x}_{-j}, \boldsymbol{C})\}]. \tag{B.2}$$

As with ordinary linear regression, ABCs identify each main effect as a group-averaged compar-

ison between expectations at $(x_j+1, \boldsymbol{x}_{-j})$ and $(x_j, \boldsymbol{x}_{-j})$. The main differences for GLMs is the presence of the link function g within this comparison.

For clarity, we provide interpretations for logistic and Poisson regression. For binary data $Y \in \{0,1\}$, $\mu(\boldsymbol{x}, \boldsymbol{c})$ equals the probability that $Y=1$ and logistic regression specifies g as the logit link, $g(t) = \log\{t/(1-t)\}$. Now, (B.1) simplifies to the log-odds-ratio:

$$g\{\mu(x_j+1, \boldsymbol{x}_{-j}, \boldsymbol{c})\} - g\{\mu(x_j, \boldsymbol{x}_{-j}, \boldsymbol{c})\} = \log\left[\frac{\text{odds}\{\mu(x_j+1, \boldsymbol{x}_{-j}, \boldsymbol{c})\}}{\text{odds}\{\mu(x_j, \boldsymbol{x}_{-j}, \boldsymbol{c})\}}\right]$$

where $\text{odds}\{\mu(\boldsymbol{x}, \boldsymbol{c})\} = \mu(\boldsymbol{x}, \boldsymbol{c})/\{1-\mu(\boldsymbol{x}, \boldsymbol{c})\}$. Thus, α_j is the group-averaged log-odds-ratio for x_j. This interpretation is natural: for the main-only logistic regression model, α_j is simply the log-odds-ratio for x_j. Similarly, for Poisson regression with $Y \in \{0, 1, \ldots, \}$, $\mu(\boldsymbol{x}, \boldsymbol{c})$ is the expectation of Y and $g(t) = \log(t)$, so (B.1) is a log-ratio:

$$g\{\mu(x_j+1, \boldsymbol{x}_{-j}, \boldsymbol{c})\} - g\{\mu(x_j, \boldsymbol{x}_{-j}, \boldsymbol{c})\} = \log\left\{\frac{\mu(x_j+1, \boldsymbol{x}_{-j}, \boldsymbol{c})}{\mu(x_j, \boldsymbol{x}_{-j}, \boldsymbol{c})}\right\}.$$

Here, α_j is the group-averaged log-ratio. Finally, we note that both of these terms involve group-averaged quantities on the log-scale. Thus, it may be more natural to consider exponentiated versions on the μ-scale, so the group-averages become weighted geometric means.

C Additional estimation invariance results

We further investigate estimation invariance for categorical main effects in the presence of categorical-categorical interactions. While Theorem 2 only included `race` and `sex` as main effects in the main-only model (6), here we additionally include continuous and other categorical main effects as in the general main-only model (1). We adopt the `race` and `sex` terminology from Example 2 and Theorem 2, but of course the results are general. For clarity, we rewrite (1) to isolate `race` and `sex` main effects as $\beta^M_{1,r}$ and $\beta^M_{2,s}$, respectively:

$$\mu^M(\boldsymbol{x}, \boldsymbol{c}) = \alpha^M_0 + \boldsymbol{x}^\top \boldsymbol{\alpha}^M + \sum_{k=1}^{K-2} \beta^M_{k,c_k} + \beta^M_{1,r} + \beta^M_{2,s}. \tag{C.1}$$

Then, as in Theorem 2, the cat-modified model adds a `race:sex` interaction:

$$\mu(\boldsymbol{x}, \boldsymbol{c}) = \alpha_0 + \boldsymbol{x}^\top \boldsymbol{\alpha} + \sum_{k=1}^{K-2} \beta_{k,c_k} + \beta_{1,r} + \beta_{2,s} + \gamma_{r,s} \tag{C.2}$$

The goal is to identify conditions under which the OLS estimates of the `race` and `sex` main effects are the same for models (C.1) and (C.2).

First, we establish some notation. Let $\boldsymbol{Z}_1$, $\boldsymbol{Z}_2$, and $\boldsymbol{Z}_{12}$ be matrix of indicators for `race`, `sex`, and `race:sex`, respectively, as defined in the proof of Theorem 2. Let $\boldsymbol{X}_0$ be the design matrix that includes an intercept, $\boldsymbol{x}$, and the matrix of indicators for each of the $K-2$ categorical variables other than `race` and `sex`. Now, $(\boldsymbol{X}_0, \boldsymbol{Z}_1, \boldsymbol{Z}_2)$ is the design matrix corresponding to (C.1) and $(\boldsymbol{X}_0, \boldsymbol{Z}_1, \boldsymbol{Z}_2, \boldsymbol{Z}_{12})$ is the design matrix corresponding to (C.2); neither has yet to incorporate identifiability constraints.

Without loss of generality, we will consider $\hat{\beta}^M_{1,r_0}$ and $\hat{\beta}_{1,r_0}$ for the r_0th `race` group; similar results will apply for the other `race` groups and any `sex` groups. Let $\hat{\boldsymbol{z}}_{1,r_0} = \boldsymbol{H}_0 \boldsymbol{z}_{1,r_0}$ be the fitted values from regressing the r_0th column of $\boldsymbol{Z}_1 = (\boldsymbol{z}_{1,1}, \ldots, \boldsymbol{z}_{1,L_R})$ on $\boldsymbol{X}_0$, so $\boldsymbol{H}_0 = \boldsymbol{X}_0(\boldsymbol{X}_0^\top \boldsymbol{X}_0)^{-1}\boldsymbol{X}_0^\top$. Finally, let $\bar{\hat{z}}_{r_0[rs]} = n_{rs}^{-1} \sum_{r_i=r, s_i=s} [\hat{\boldsymbol{z}}_{1,r_0}]_i$ be the sample mean of these fitted values within `race` group r and `sex` group s.

Under ABCs, the following condition ensures estimation invariance for the r_0th `race` group main effect:

$$\bar{\hat{z}}_{r_0[rs]} = k_0^{r_0} + k_{1,r}^{r_0} + k_{2,s}^{r_0} \tag{C.3}$$

where $k_0^{r_0}$, $k_{1,r}^{r_0}$, and $k_{2,s}^{r_0}$ are any constants, $r = 1, \ldots, L_R$, $s = 1, \ldots, L_S$. We present the main result and the proof, and then discuss the condition (C.3) subsequently.

Theorem 8. *Under ABCs* (8a) *and* (10) *and condition* (C.3)*, the OLS estimates of the main effects under the main-only model* (C.1) *and the cat-modified model* (C.2) *satisfy* $\hat{\beta}^M_{1,r_0} = \hat{\beta}_{1,r_0}$.

Theorem 8 can be applied for any individual `race` main effect or aggregated across all `race` coefficients by enumerating condition (C.3) for $r_0 = 1, \ldots, L_R$. Modifications to include `sex` main effects proceed in the same way by replacing $\boldsymbol{z}_{r_0}$ with columns of $\boldsymbol{Z}_2$. When the continuous covariates are centered ($\bar{\boldsymbol{x}} = \boldsymbol{0}$), we also obtain estimation invariance for the intercept, $\hat{\alpha}_0^M = \hat{\alpha}_0 = \bar{y}$, by Theorem 1. Extensions for inference invariance follow the same arguments as in Section 3.2.

Proof (Theorem 8). As summarized in (A.2), a sufficient condition for estimation invariance is orthogonality under the ABCs reparametrization of the categorical-categorical interaction: $(\boldsymbol{z}_{1,r_0} - \boldsymbol{H}_0 \boldsymbol{z}_{1,r_0})^\top \boldsymbol{Z}_{12} \boldsymbol{Q}_{\hat{\boldsymbol{\pi}}} = \boldsymbol{0}$, where $\boldsymbol{Q}_{\hat{\boldsymbol{\pi}}}$ is defined in the proof of Theorem 2. That proof established three key results under ABCs (10): (i) $\boldsymbol{1}^\top \boldsymbol{Z}_{12} \boldsymbol{Q}_{\hat{\boldsymbol{\pi}}} = \boldsymbol{0}$, (ii) $\boldsymbol{Z}_1^\top \boldsymbol{Z}_{12} \boldsymbol{Q}_{\hat{\boldsymbol{\pi}}} = \boldsymbol{0}$, and (iii) $\boldsymbol{Z}_2^\top \boldsymbol{Z}_{12} \boldsymbol{Q}_{\hat{\boldsymbol{\pi}}} = \boldsymbol{0}$, each with zeros of the appropriate dimensions. Recall that $\boldsymbol{Z}_1 = (\boldsymbol{z}_{1,1}, \ldots, \boldsymbol{z}_{1,L_R})$ and similarly define $\boldsymbol{Z}_2 = (\boldsymbol{z}_{2,1}, \ldots, \boldsymbol{z}_{2,L_S})$, so that $\boldsymbol{z}_{1,r}$ and $\boldsymbol{z}_{2,s}$ are the n-dimensional indicator

vectors for `race` $= r$ and `sex` $= s$, respectively. Applying (ii), it is clear that $\boldsymbol{z}_{1,r_0}^\top \boldsymbol{Z}_{12} \boldsymbol{Q}_{\hat{\boldsymbol{\pi}}} = \mathbf{0}$ so it only remains to show that $\hat{\boldsymbol{z}}_{1,r_0}^\top \boldsymbol{Z}_{12} \boldsymbol{Q}_{\hat{\boldsymbol{\pi}}} = \mathbf{0}$. Observe that for any vector $\boldsymbol{u}$ of appropriate length, $\boldsymbol{u}^\top \boldsymbol{Z}_{12} = (\cdots n_{rs} \bar{u}_{[rs]} \cdots)$ where $n_{rs} = \sum_{i=1}^n \mathbb{I}(r_i = r, s_i = s) = n\hat{\pi}_{rs}$ are the totals by `race` $= r$ and `sex` $= s$. Then applying condition (C.3),

$$
\begin{aligned}
\hat{\boldsymbol{z}}_{1,r_0}^\top \boldsymbol{Z}_{12} &= (n_{11} \bar{\hat{z}}_{r_0[11]}, \ldots, n_{rs} \bar{\hat{z}}_{r_0[rs]}, \ldots, n_{L_R L_S} \bar{\hat{z}}_{r_0[L_R L_S]}) \\
&= \{n_{11}(k_0^{r_0} + k_{1,1}^{r_0} + k_{2,1}^{r_0}), \ldots, n_{rs}(k_0^{r_0} + k_{1,r}^{r_0} + k_{2,s}^{r_0}), \ldots, n_{L_R L_S}(k_0^{r_0} + k_{1,L_R}^{r_0} + k_{2,L_S}^{r_0}\}.
\end{aligned}
$$

Observing that each entry (r, s) may be written

$$
n_{rs}(k_0^{r_0} + k_{1,r}^{r_0} + k_{2,s}^{r_0}) = n_{rs} k_0^{r_0} + \sum_{r'=1}^{L_R} n_{rs} k_{1,r'}^{r_0} \mathbb{I}\{r' = r\} + \sum_{s'=1}^{L_S} n_{rs} k_{2,s'}^{r_0} \mathbb{I}\{s' = s\}
$$

and recalling the definitions of $\boldsymbol{z}_{1,r}$ and $\boldsymbol{z}_{2,s}$, we obtain the key representation in vector form,

$$
\hat{\boldsymbol{z}}_{1,r_0}^\top \boldsymbol{Z}_{12} = k_0^{r_0} \mathbf{1}^\top \boldsymbol{Z}_{12} + \sum_{r=1}^{L_R} k_{1,r}^{r_0} \boldsymbol{z}_{1,r}^\top \boldsymbol{Z}_{12} + \sum_{s=1}^{L_S} k_{2,s}^{r_0} \boldsymbol{z}_{2,s}^\top \boldsymbol{Z}_{12}.
$$

Post-multiplying by $\boldsymbol{Q}_{\hat{\boldsymbol{\pi}}}$ and applying (i), (ii), and (iii) zeros out each term, proving the result. □

To understand the condition (C.3), recall that $\hat{\boldsymbol{z}}_{1,r_0}$ contains the fitted values from $\boldsymbol{X}_0$ for the r_0th `race` group indicators as in a linear probability model. Condition (C.3) allows these fitted values, once aggregated by `race` and `sex`, to vary additively by `race` and `sex`. As a special case, consider Theorem 2 where $\boldsymbol{X}_0 = \mathbf{1}_n$ only includes the intercept. Now, the fitted values are $\hat{\boldsymbol{z}}_{r_0} = \hat{\pi}_{r_0} \mathbf{1}_n$ and condition (C.3) applies with $k_0^{r_0} = \hat{\pi}_{r_0}$ and $k_{1,r}^{r_0} = k_{2,s}^{r_0} = 0$. Since this is true for all `race` and `sex` groups, Theorem 8 implies Theorem 2. This example also shows the importance of allowing the associations in condition (C.3) to be specific to the r_0th `race` group under consideration. For a different interpretation, condition (C.3) can be modified to use the residuals $\boldsymbol{z}_{r_0} - \hat{\boldsymbol{z}}_{r_0}$ instead of the fitted values $\hat{\boldsymbol{z}}_{1,r_0}$ and Theorem 8 will still hold.

For practical applications with general $\boldsymbol{X}_0$, we view condition (C.3) as an approximation that leads to approximate estimation invariance. To clarify this point, consider a cat-modified two-way ANOVA for the fitted values, $\hat{\boldsymbol{z}}_{1,r_0} \sim$ `race + sex + race:sex`. Condition (C.3) holds whenever the OLS estimates of the `race:sex` coefficients are identically zero. Thus, the accuracy of the approximation $\hat{\beta}_{1,r_0}^M \approx \hat{\beta}_{1,r_0}$ is directly linked to the magnitude of these `race:sex` coefficients. Although these coefficients will not equal zero in general, large values

would require that the predictions by $\boldsymbol{X}_0$ for the r_0th `race` group (or their residuals) not only vary by `race` and `sex`, but do so in a strongly interactive way. In our real data application with multiple continuous and categorical variables—and strong dependencies among them—we find that Theorem 8 is highly accurate (Figure 6, bottom left).

To investigate this numerically, we simulate data as in Section 4.2. The simulated data include dependent `race` and `sex` variables along with $p = 10$ continuous covariates, half of which are correlated with `race`. We set $n = 500$. No other specifications are required here; the computations below involve only the continuous and categorical variables, so there is no need to define model parameters or generate $\boldsymbol{y}$.

We examine the quantity $(\boldsymbol{Z}_1 - \boldsymbol{H}_0\boldsymbol{Z}_1)^\top \boldsymbol{Z}_{12}$ and variations that account for identifiability constraints, similar to (A.1) and (A.2). Estimation invariance of the `race` main effects corresponds to exact zeros. We compute squared Frobenius norms for four versions:

$$\begin{aligned}
\|(\boldsymbol{Z}_1 - \boldsymbol{H}_0\boldsymbol{Z}_1)^\top \boldsymbol{Z}_{12}\|_F^2 &= 13757.41 \\
\|(\boldsymbol{Z}_1 - \boldsymbol{H}_0\boldsymbol{Z}_1)^\top \boldsymbol{Z}_{12}^{RGE}\|_F^2 &= 4291.74 \\
\|(\boldsymbol{Z}_1 - \boldsymbol{H}_0\boldsymbol{Z}_1)^\top \boldsymbol{Z}_{12}^{STZ}\|_F^2 &= 895.34 \\
\|(\boldsymbol{Z}_1 - \boldsymbol{H}_0\boldsymbol{Z}_1)^\top \boldsymbol{Z}_{12}^{ABC}\|_F^2 &= 5.25.
\end{aligned}$$

The first does not include any identifiability constraints for the categorical-categorical interaction. The second uses RGE for the categorical-categorical interaction, so $\boldsymbol{Z}_{12}^{RGE}$ drops the columns of $\boldsymbol{Z}_{12}$ corresponding to the `race` and `sex` reference groups adopted in Section 4.2. The third uses STZ constraints, with $\boldsymbol{Z}_{12}^{STZ}$ constructed in the same way as $\boldsymbol{Z}_{12}^{ABC}$ but with $\hat{\boldsymbol{\pi}} \propto \mathbf{1}$. Finally, $\boldsymbol{Z}_{12}^{ABC} = \boldsymbol{Z}_{12}\boldsymbol{Q}_{\hat{\boldsymbol{\pi}}}$ uses ABCs. Clearly, the version with ABCs is much closer to zero—and thus closer to estimation (and inference) invariance for the `race` main effects—even though condition (C.3) is not enforced.

D Additional simulation results

First, we revisit the analysis from Section 4.1, but now for categorical-continuous interactions: given categorical `race` and continuous `x`, what is the effect of including the `x:race` interaction on the main x-effect? The theory of ABCs (Section 3) predicts that invariance for estimation and inference is contingent on the equal-variance condition (13). We investigate the sensitivity to this condition as well as to the magnitude of the interaction effect.

To incorporate dependencies between `race` and `x`, we simulate `race` as in Section 4.1 and then simulate `x` conditional on `race`:

$$[\texttt{x} \mid \texttt{race} = r] \sim \begin{cases} 5 + \sigma_{ac} N(0,1) & r = \texttt{A} \\ \sqrt{12}\ \text{Uniform}(0,1) & r = \texttt{B} \\ -5 + \sigma_{ac} t_8(0,1) & r = \texttt{C} \\ \text{Gamma}(1,1) & r = \texttt{D} \end{cases} \tag{D.1}$$

Each `race` group features a unique distribution with varying means, so `x` and `race` are strongly dependent and highly correlated. Here, σ_{ac} controls the degree to which the equal-variance condition (13) is violated: $\sigma_{ac} = 1$ is a mild violation (the race-specific *population* variances are identical, but the sample quantities $\hat{\sigma}^2_{x[r]}$ are not) while $\sigma_{ac} = 1.5$ is a strong violation. The response variable y is simulated with expectation (5) with $\alpha_0 = \alpha_1 = 1$, $\beta_c = -1$, and $\gamma_b = \gamma$, and all other coefficients zero, or equivalently, $\mu(x, r) = 1 + x - \mathbb{I}\{r = \texttt{C}\} + \gamma x \mathbb{I}\{r = \texttt{B}\}$ plus $t_4(0,1)$-distributed errors. This data-generating process satisfies RGE ($\beta_a = 0$), but not ABCs, and includes non-Gaussian errors. Again, $\gamma \in \{0, 0.5, 1.5\}$ determines the magnitude of the interaction effect. We repeat this process to create 500 synthetic datasets.

For each simulated dataset, we fit the main-only model (4) and the cat-modified model (5) and compare the estimates and SEs for main x-effect α_1 between the two models under ABCs, RGE, and STZ. The estimates are in Figure D.1. Even with mild deviations from the equal-variance condition (13), the x-effect estimates under ABCs are nearly identical between models that do and do not include the `x:race` interaction. Crucially, this invariance persists regardless of the true interaction effect magnitude γ. Under strong violations of (13) *and* a strong interaction effect, Theorem 3 no longer applies. However, this behavior is reasonable: when (13) is strongly violated, a one-unit change in x is not comparable for different `race` groups, so only the model that includes race-specific x-effects (via the `x:race` interaction) is appropriate. Finally, we note the absence of invariance for estimation with RGE or STZ. These estimators change dramatically when γ is moderate to large. Even when $\gamma = 0$—when classical consistency results for OLS should provide asymptotic invariance in this case—they do not match the invariance of ABCs.

The SEs are in Figure D.2. As long as the violations of (13) are mild ($\sigma_{ac} = 1$), the SEs of the x-effect, under ABCs, are 1) nearly identical between the main-only and cat-modified models when the true interaction effect is small and 2) smaller for the cat-modified model when

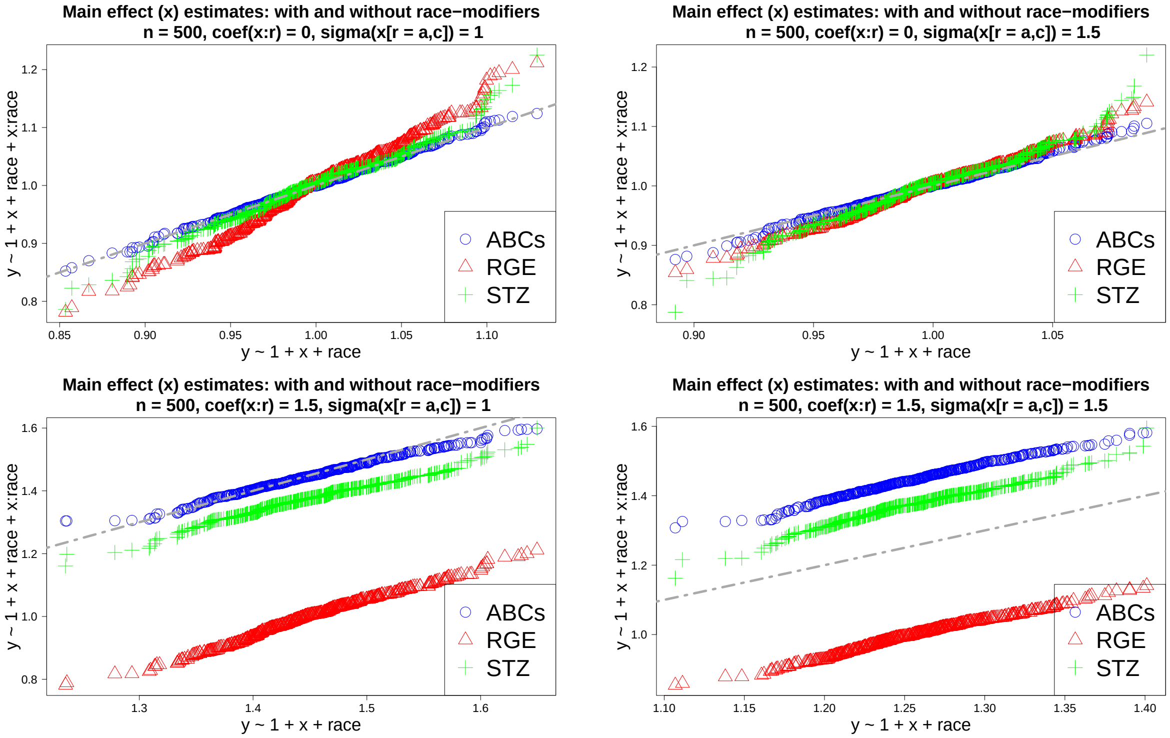

Figure D.1: Estimates for the main x-effect for models that do (y-axis) and do not (x-axis) include the `x:race` interaction across 500 simulated datasets. Under ABCs, the estimates are nearly invariant ($45°$ line) as long as the deviations from equal-variance (13) are mild ($\sigma_{ac} = 1$, left), regardless of whether the true interaction effect is zero ($\gamma = 0$, top) or large ($\gamma = 1.5$, bottom). When γ is large *and* (13) is strongly violated (bottom right), ABCs no longer offer invariance under Theorem 3. RGE and STZ offer no such invariance and depend critically on γ.

the true interaction effect is large. Critically, including the `x:race` interaction *under ABCs* does not sacrifice any statistical power for the main x-effect, and in some cases enhances it. This is decisively not the case for RGE or STZ: when the true interaction effect is zero, adding the `x:race` interaction decreases statistical power for the main x-effect.

The same caveats and conclusions about the "main effects" from Section 4.1 apply: RGE, STZ, and ABCs are targeting different functionals of $\mu(x, r)$, but again we argue that the estimation and inference properties of the "main effects" are most ideal under ABCs.

Next, we expand upon the results from Section 4.1 and those above to consider moderate interaction effects ($\gamma = 0.5$). The results are in Figures D.3 and D.4, respectively.

Finally, we expand upon the results from Section 4.2 to consider smaller ($n = 200$) and larger ($n = 1000$) sample sizes (see Figures D.5 and D.6) along with predictive evaluations based on RMSEs for $\mu(\boldsymbol{x}, \boldsymbol{c})$ that compare ABCs and RGE for lasso and ridge regression, also including an "overparametrized" version that does not impose any constraint (Figure D.7).

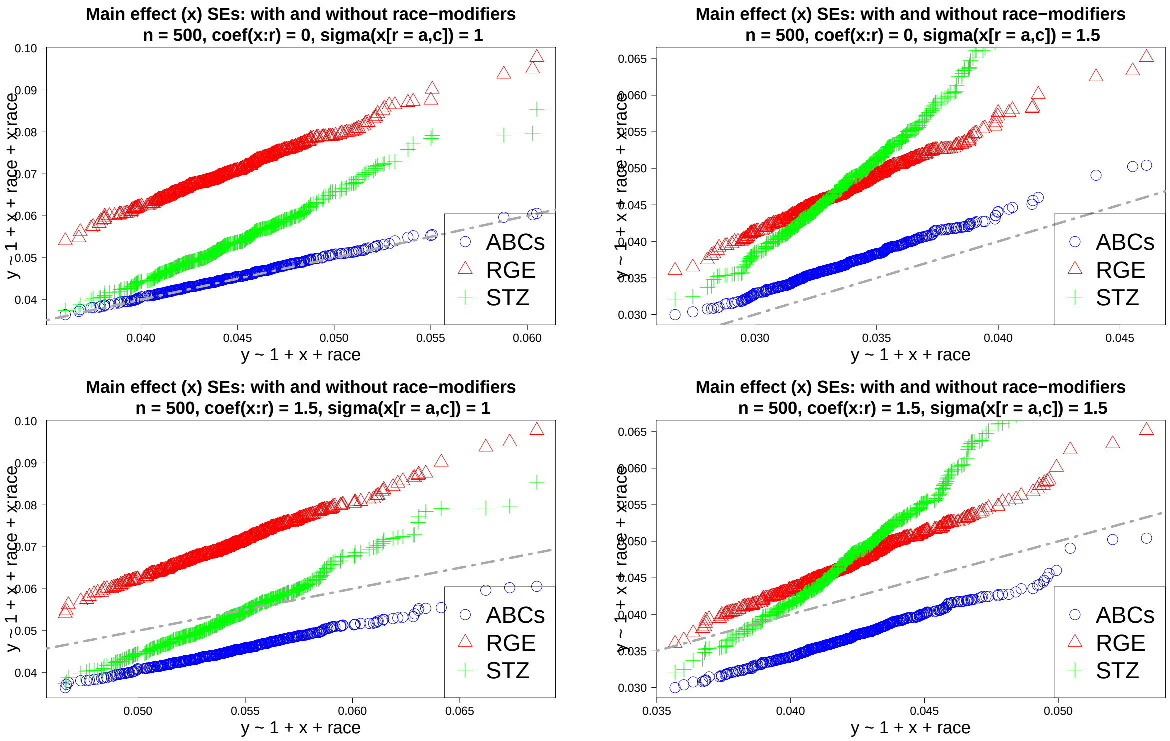

Figure D.2: Standard errors (SEs) for the main x-effect for models that do (y-axis) and do not (x-axis) include the `x:race` interaction across 500 simulated datasets. Under ABCs, the SEs are nearly identical between the two models (45° line) when the true interaction effect is zero *and* deviations from equal-variance (13) are mild ($\gamma = 0$, $\sigma_{ac} = 1$, top left). If instead the interaction effect is large ($\gamma = 1.5$, $\sigma_{ac} = 1$, bottom left), the SEs under ABCs reduce substantially (by about 15%) for the model that includes the `x:race` interaction. These effects are not assured when (13) is strongly violated ($\sigma_{ac} = 1.5$, right). All results are consistent with Theorem 7. Similar properties do *not* occur for RGE or STZ, regardless of γ and σ_{ac}.

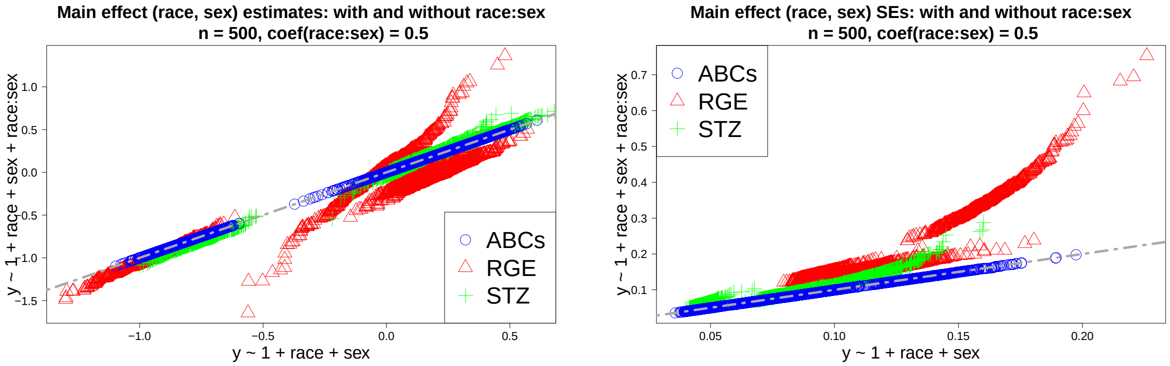

Figure D.3: Estimates (left) and standard errors (SEs, right) for all `race` and `sex` main effects for models that do (y-axis) and do not (x-axis) include the `race:sex` interaction across 500 simulated datasets. Here, the interaction effect is moderate ($\gamma = 0.5$). Under ABCs, the estimates are exactly invariant and the SEs are nearly invariant (45° line).

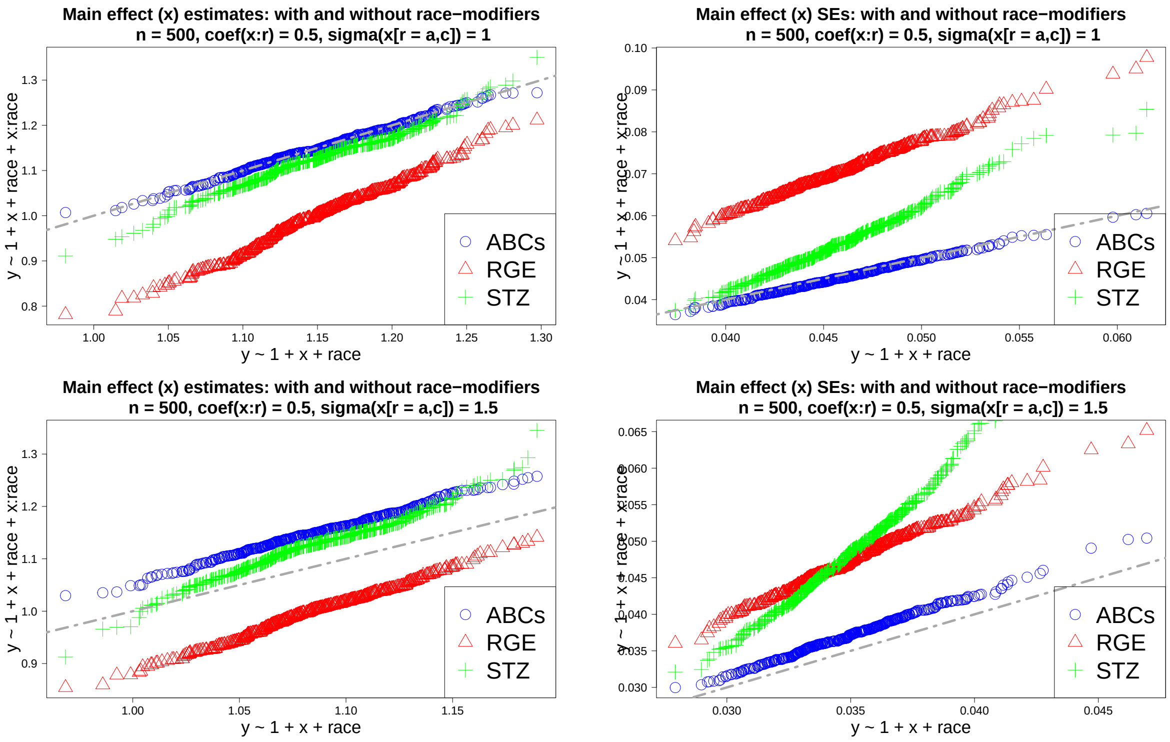

Figure D.4: Estimates (left) and standard errors (SEs, right) for the main x-effect for models that do (y-axis) and do not (x-axis) include the `x:race` interaction across 500 simulated datasets. Here, the interaction effect is moderate ($\gamma = 0.5$) in all cases. Under ABCs, the estimates and SEs are nearly invariant (45° line) as long as the deviations from equal-variance (13) are mild ($\sigma_{ac} = 1$, top). These effects are not assured when (13) is strongly violated (bottom).

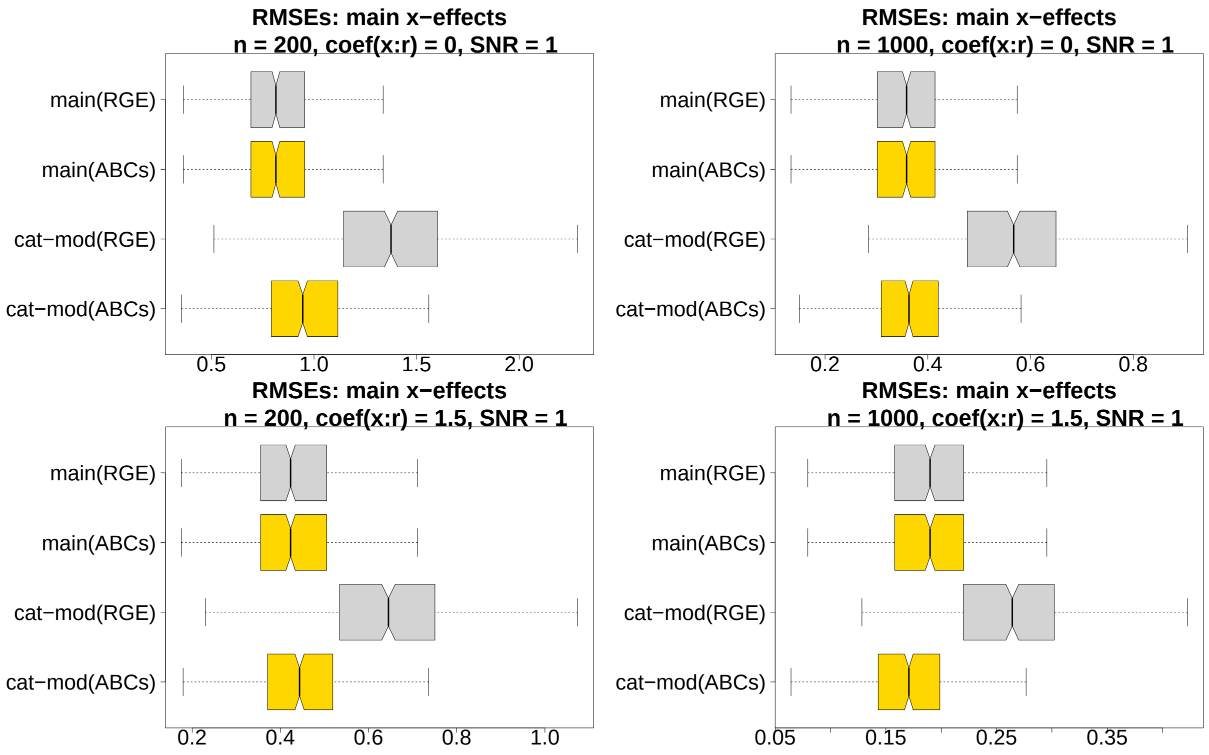

Figure D.5: RMSEs for the main x-effects with extraneous (top) or necessary (bottom) cat-modifier effects for $n = 200$ (left) and $n = 1000$ (right) under main-only and cat-modified models with ABCs (gold) and RGE (gray). Boxplots are across 500 simulations; nonoverlapping notches indicate a difference in medians. For $n = 200$, the cat-modified models omit the `race:sex` interaction to avoid rank deficiency. For larger n, the cat-modified model with ABCs is better able to match (top right) or improve upon (bottom right) the main x-effect estimates compared to the main-only models.

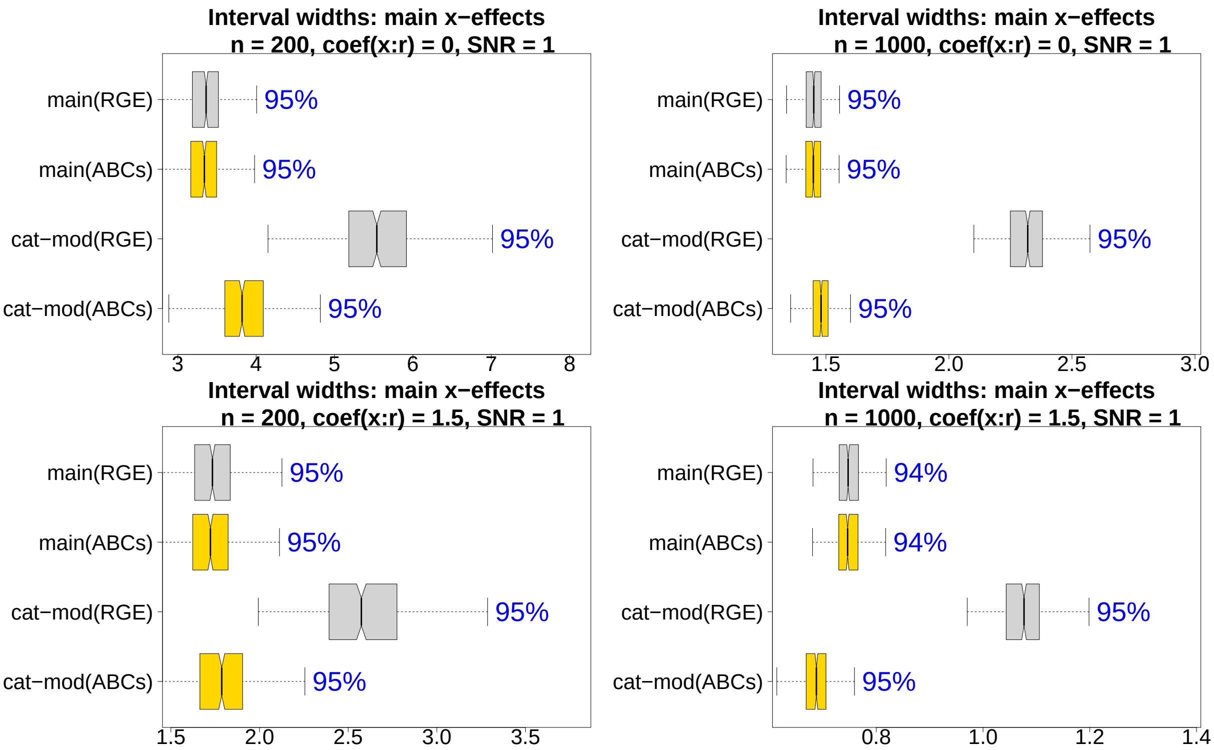

Figure D.6: Interval widths (boxplots) and empirical coverage (annotations) for 95% confidence intervals for the main x-effects with extraneous (top) or necessary (bottom) cat-modifier effects for $n = 200$ (left) and $n = 1000$ (right) under main-only and cat-modified models with ABCs (gold) and RGE (gray). For $n = 200$, the cat-modified models omit the `race:sex` interaction to avoid rank deficiency. For larger n, the cat-modified model with ABCs is better able to match (top right) or improve upon (bottom right) the statistical power for the main x-effects compared to the main-only models.

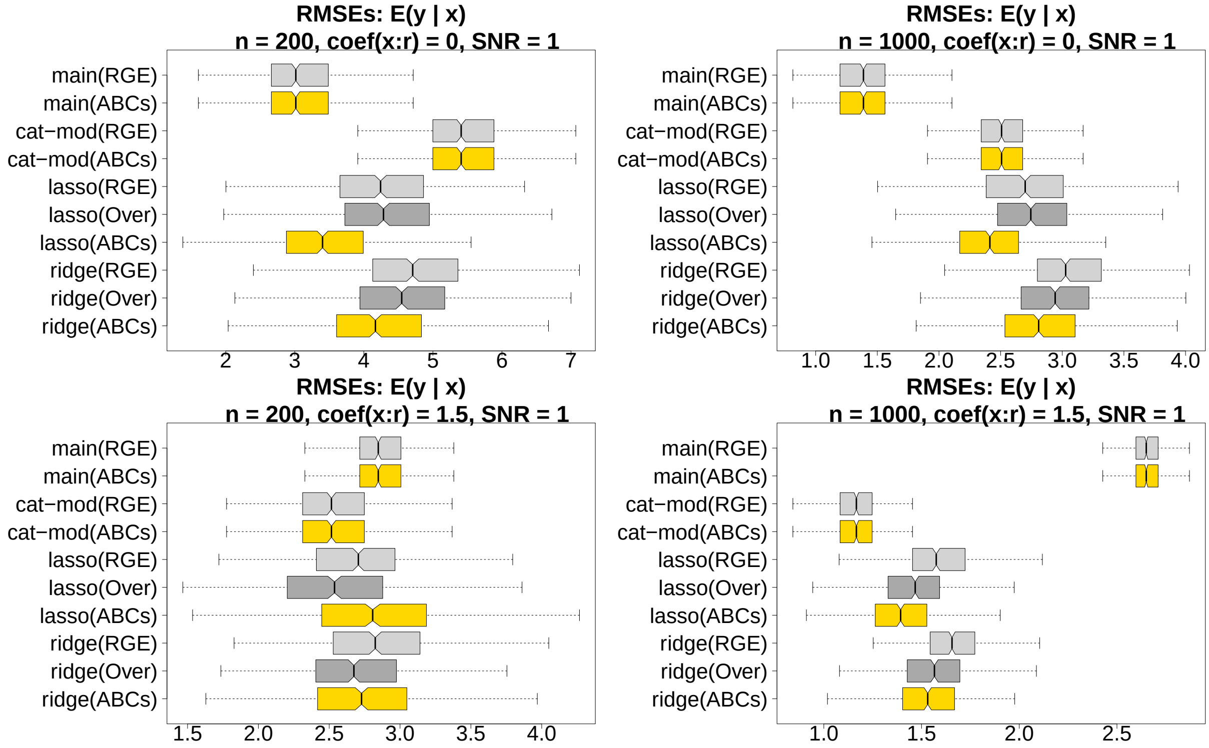

Figure D.7: RMSEs for prediction of $\mu(\boldsymbol{x}, r, s)$ with extraneous (top) or necessary (bottom) cat-modifier effects for $n = 200$ (left) and $n = 1000$ (right) under main-only and cat-modified models with ABCs (gold) and RGE (gray). Boxplots are across 500 simulations; nonoverlapping notches indicate a difference in medians. All lasso and ridge estimators use the cat-modified model. Predictions under OLS are identical between RGE and ABCs. For $n = 200$, the cat-modified models omit the `race:sex` interaction to avoid rank deficiency. For each penalized (lasso or ridge) regression, ABCs typically outperform both RGE and the overparametrized models that omits any constraints.

E Additional application details

Reference group encoding (RGE)				Abundance-based constraints (ABCs)			
Variable	Model	Estimate (SE)	p-value	Variable	Model	Estimate (SE)	p-value
`Intercept`	Main-only	0.238 (0.010)	<0.001	`Intercept`	Main-only	0.000 (0.006)	1.000
	Cat-modified	0.217 (0.011)	<0.001		Cat-modified	0.000 (0.006)	1.000
`White`	Main-only	ref	ref	`White`	Main-only	0.256 (0.005)	<0.001
	Cat-modified	ref	ref		Cat-modified	0.256 (0.005)	<0.001
`Black`	Main-only	-0.727 (0.013)	<0.001	`Black`	Main-only	-0.471 (0.008)	<0.001
	Cat-modified	-0.664 (0.018)	<0.001		Cat-modified	-0.471 (0.008)	<0.001
`Hispanic`	Main-only	-0.016 (0.025)	0.517	`Hispanic`	Main-only	0.240 (0.023)	<0.001
	Cat-modified	-0.042 (0.035)	0.228		Cat-modified	0.240 (0.023)	<0.001
`Female`	Main-only	ref	ref	`Female`	Main-only	-0.018 (0.006)	0.003
	Cat-modified	ref	ref		Cat-modified	-0.018 (0.006)	0.003
`Male`	Main-only	0.036 (0.012)	0.003	`Male`	Main-only	0.018 (0.006)	0.003
	Cat-modified	0.077 (0.015)	<0.001		Cat-modified	0.018 (0.006)	0.003
`White:Female`	Cat-modified	ref	ref	`White:Female`	Cat-modified	-0.021 (0.005)	<0.001
`Black:Female`	Cat-modified	ref	ref	`Black:Female`	Cat-modified	0.043 (0.008)	<0.001
`Hisp:Female`	Cat-modified	ref	ref	`Hisp:Female`	Cat-modified	-0.046 (0.022)	0.034
`White:Male`	Cat-modified	ref	ref	`White:Male`	Cat-modified	0.021 (0.005)	<0.001
`Black:Male`	Cat-modified	-0.128 (0.025)	<0.001	`Black:Male`	Cat-modified	-0.044 (0.008)	<0.001
`Hisp:Male`	Cat-modified	0.056 (0.050)	0.262	`Hisp:Male`	Cat-modified	0.051 (0.024)	0.034

Table E.1: Linear regression output with RGE (left) and ABCs (right) for the main-only model (6) and the cat-modified model (7) for the North Carolina education data (Section 5). The (mother's) race groups are non-Hispanic White (58.7%), non-Hispanic Black (35.1%), and Hispanic (6.2%) and the child's sex are `Female` (50.1%) and `Male` (49.9%). With RGE (references `White` and `Female`), the main effects change dramatically with the addition of cat-modifiers and the standard errors (SEs) uniformly inflate. Yet with ABCs, all main effect estimates *and* SEs are invariant to cat-modifiers (the SEs actually decrease slightly; this is obscured due to rounding).

Variable j	$\hat\sigma_{x[\texttt{NHW}]}(j)$	$\hat\sigma_{x[\texttt{NHB}]}(j)$	$\hat\sigma_{x[\texttt{Hisp}]}(j)$
Racial isolation (RI)	0.691	1.071	0.942
Blood lead level	0.951	1.042	0.977
Birthweight percentile for gestational age	0.994	0.963	0.979
Mother's age	0.999	0.971	0.889
$\text{PM}_{2.5}$ exposure	0.998	1.005	0.928

Table E.2: The (scaled) sample standard deviations $\hat\sigma_{x[r]}(j)$ by race r for each covariate $j = 1, \ldots, p$. The invariance result for estimators with and without cat-modifiers (Theorem 4) requires $\hat\sigma_{x[\texttt{NHW}]}(j) = \hat\sigma_{x[\texttt{NHB}]}(j) = \hat\sigma_{x[\texttt{Hisp}]}(j)$ for each covariate j (and similarly for the cross-covariances). Although this condition is clearly violated, the estimates and SEs maintain invariance, which suggests strong empirical robustness for the desirable invariance property of ABCs.

Variable (continued)	Estimate (SE)	p-value
Economically disadvantaged (EconDisadv)		
No (39.5%)	0.163 (0.009)	<0.001
Yes (60.5%)	-0.106 (0.006)	<0.001
White:EconDisadvNo	0.010 (0.004)	0.018
Black:EconDisadvNo	-0.034 (0.023)	0.138
Hisp:EconDisadvNo	-0.171 (0.063)	0.007
White:EconDisadvYes	-0.013 (0.006)	0.018
Black:EconDisadvYes	0.007 (0.005)	0.138
Hisp:EconDisadvYes	0.025 (0.009)	0.007
EconDisadvNo:Male	-0.013 (0.008)	0.118
EconDisadvYes:Male	0.009 (0.006)	0.118
EconDisadvNo:Female	0.014 (0.009)	0.118
EconDisadvYes:Female	-0.009 (0.006)	0.118
EconDisadvNo:mEdu<HS	-0.056 (0.037)	0.126
EconDisadvYes:mEdu<HS	0.006 (0.004)	0.126
EconDisadvNo:mEdu=HS	-0.039 (0.012)	0.002
EconDisadvYes:mEdu=HS	0.016 (0.005)	0.002
EconDisadvNo:mEdu>HS	0.020 (0.005)	<0.001
EconDisadvYes:mEdu>HS	-0.043 (0.011)	<0.001
RI:EconDisadvNo	-0.007 (0.011)	0.513
RI:EconDisadvYes	0.005 (0.007)	0.513
BLL:EconDisadvNo	-0.011 (0.009)	0.229
BLL:EconDisadvYes	0.007 (0.006)	0.229
BWTpct:EconDisadvNo	0.000 (0.009)	0.983
BWTpct:EconDisadvYes	0.000 (0.006)	0.983
mAge:EconDisadvNo	0.016 (0.009)	0.088
mAge:EconDisadvYes	-0.011 (0.006)	0.088
PM2.5:EconDisadvNo	0.010 (0.009)	0.230
PM2.5:EconDisadvYes	-0.007 (0.006)	0.230

Table E.3: Cat-modified model output under ABCs for NC STEM education outcomes. These results augment Table 2 to include EconDisadv main and interaction effects, where "Economically disadvantaged" is determined by participation in the National Lunch Program. EconDisadv is associated with lower math scores and eliminates the significant positive benefits of higher-educated mothers (mEdu>HS), thus emphasizing the importance of heterogeneous effects.
